# Sex-Stratified Transcriptomic Analysis of Cord-Blood Endothelial Colony-Forming Cells from Pregnancies Complicated by Gestational Diabetes

**DOI:** 10.34133/csbj.0252

**Published:** 2026-11-06

**Authors:** Muyiwa S. Adegbaju, Oluseyi Babayeju, Olanrewaju B. Morenikeji, Olusola Ojurongbe, Bolaji N. Thomas

**Affiliations:** ^1^ Department of Biomedical Sciences, Rochester Institute of Technology, Rochester, NY, USA.; ^2^ School of Medicine, Newcastle University, Newcastle upon Tyne, UK.; ^3^ Department of Biology, Virginia State University, Petersburgh, VA, USA.; ^4^ Department of Medical Microbiology and Parasitology, Ladoke Akintola University of Technology, Ogbomosho, Nigeria.

## Abstract

Gestational diabetes mellitus (GDM) and maternal obesity are intrauterine metabolic exposures associated with altered fetal development and increased cardiovascular risk later in life. Increasing evidence suggests that fetal responses to these exposures are sex-dependent; however, the regulatory mechanisms underlying sex-specific vascular programming remain poorly understood. Using purified neonatal endothelial colony-forming cells (ECFCs), a progenitor population of fetal vasculature, we performed a sex-stratified transcriptomic analysis of pregnant mothers classified as lean, overweight, or GDM. Differential expression, pathway enrichment, transcription factor activity inference, kinase network reconstruction, and connectivity mapping were integrated to characterize sex-dependent regulatory responses to maternal metabolic dysfunction. Male and female ECFCs exhibited markedly divergent transcriptomic responses to GDM. Correlation between male and female fold-change estimates was negligible (*r* = −0.01), and the female-specific signal was not detectable when sex was modeled as a covariate rather than as a stratifying variable. Female GDM-exposed ECFCs demonstrated coordinated enrichment of cell-cycle- and DNA replication-associated pathways together with recurrent prioritization of E2F-family regulators across multiple analytical approaches. In contrast, the male response was comparatively limited and was associated with distinct transcriptional changes, including *AKT3*-associated pathways. Notably, *E2F1* transcript abundance remained relatively stable despite coordinated expression changes among E2F-associated target genes. Independent endothelial transcriptomic datasets, drawn from previously published findings rather than independent reanalysis, provided complementary biological context rather than direct validation, consistent with enrichment of E2F-associated programs in GDM-exposed ECFCs and greater transcriptional responsiveness in female endothelial cells reported elsewhere. These findings indicate that fetal sex is a major determinant of the endothelial progenitor response to maternal metabolic dysfunction and that biologically relevant signals may be obscured in pooled analyses. Our results support the existence of sex-dependent regulatory programs associated with fetal vascular responses to GDM and identify an E2F1-associated regulatory framework as a candidate mechanism warranting further investigation.

## Introduction

Gestational diabetes mellitus (GDM), often occurring alongside maternal obesity, represents a major intrauterine metabolic exposure associated with increased cardiovascular and reproductive health risks in offspring later in life [1]. These associations are commonly interpreted within the Developmental Origins of Health and Disease (DOHaD) framework, which proposes that environmental conditions during critical developmental windows can induce lasting structural and functional changes in fetal tissues and thereby influence disease susceptibility across the lifespan. The conceptual foundations of this framework can be traced to the “thrifty phenotype” model, which proposed that poor fetal and early-life nutrition may induce persistent developmental adaptations that increase the risk of metabolic disease in adulthood [2,3]. The field has since expanded considerably and now encompasses a broad range of intrauterine exposures, including maternal obesity and hyperglycemia. Within this broader framework, maternal hyperglycemia and hyperinsulinemia may induce persistent structural and functional alterations within the feto-placental unit during critical periods of development [4].

A growing body of evidence indicates that fetal programming is not a sex-neutral process. Studies of the human placenta and fetus across diverse maternal conditions, including asthma [5,6], pre-eclampsia [7,8], and preterm delivery [9,10], have demonstrated that male and female offspring often exhibit distinct physiological and developmental responses to a shared intrauterine environment. These observations have led to the broader concept that fetal sex influences developmental adaptation to maternal stressors, although the direction and magnitude of these responses vary according to the nature of the exposure [11]. More broadly, sex-specific responses to metabolic stress are increasingly recognized across endocrine and metabolic disorders, with pathways such as estrogen–MAPK (mitogen-activated protein kinase) signaling proposed as contributors to divergent biological outcomes between males and females [12]. This sex dependence extends to the transcriptomic level. For example, a recent sex-stratified RNA-sequencing (RNA-seq) study of term placentas reported 150 differentially expressed genes in male-bearing placentas from pregnancies complicated by late-onset pre-eclampsia, compared with none in female-bearing placentas, a difference that became apparent only after stratification by fetal sex [13]. Consistent with this principle, accumulating evidence indicates that GDM-associated transcriptional alterations occur more extensively in female progeny than in males, despite exposure to a broadly similar intrauterine metabolic environment [14,15]. Together, these findings suggest that fetal sex can influence both the magnitude and the regulatory organization of responses to maternal metabolic dysfunction and support the need for sex-stratified, rather than sex-adjusted, investigation of fetal vascular cell populations that may retain molecular evidence of developmental programming.

Although increasing evidence supports sex-dependent molecular responses to maternal metabolic dysfunction, identifying the cellular basis of these adaptations remains challenging. A major obstacle is the cellular heterogeneity of placental tissue, where transcriptomic profiles represent composite signals from multiple cell populations and may therefore obscure cell-type-specific responses to maternal metabolic stress [15]. Endothelial colony-forming cells (ECFCs), isolated from neonatal cord blood, provide a valuable model for investigating fetal vascular biology within a relatively homogeneous endothelial progenitor population [16]. Previous studies have shown that exposure to gestational diabetes and excessive maternal weight gain is associated with impaired ECFC function, including reduced self-renewal capacity, delayed colony outgrowth, and impaired wound-healing responses [17,18]. These findings indicate that adverse intrauterine metabolic conditions can have lasting effects on fetal vascular progenitors.

Despite evidence that maternal metabolic dysfunction impairs ECFC function, the molecular basis of these phenotypes remains poorly understood. Previous studies have focused on describing transcriptional differences or cellular phenotypes rather than investigating the regulatory networks that may organize these responses. Consequently, it remains unclear whether sex-dependent vascular adaptation to maternal metabolic dysfunction reflects distinct transcriptional regulatory programs or quantitative variation within a shared biological response. An important feature of the ECFC model is that these cells are isolated, cultured, and passaged in vitro in the absence of an ongoing maternal signal. Transcriptional alterations detected under these conditions therefore reflect persistent differences in cellular state rather than acute responses to the intrauterine environment. Such persistence is consistent with the concept of developmental programming as a lasting alteration in cellular structure or regulatory function induced during a critical developmental window. However, the present study examined molecular features of programming and did not assess downstream clinical outcomes in offspring.

We hypothesized that male and female ECFCs engage distinct transcriptional responses to maternal metabolic dysfunction that are organized through divergent upstream regulatory networks. This hypothesis extends the established principle that fetal sex shapes developmental adaptation to maternal stressors to a new exposure (GDM) and a previously uncharacterized tissue compartment (fetal vascular progenitors). To investigate this possibility, we performed a sex-stratified transcriptomic analysis of purified neonatal ECFCs derived from lean, overweight, and gestational diabetes pregnancies. By integrating differential expression analysis with pathway enrichment, rank–rank hypergeometric overlap (RRHO), transcription factor activity inference, kinase network reconstruction, and connectivity mapping, we sought to define the regulatory architecture associated with sex-dependent endothelial responses to adverse intrauterine metabolic exposure. Through this systems-level framework, we aim to identify candidate regulatory mechanisms that may contribute to a sexually dimorphic vascular programming in offspring exposed to gestational diabetes.

## Materials and Methods

### Data source and *in silico* verification of cell identity

Transcriptomic profiles were obtained from neonatal ECFCs (*n* = 60) previously isolated and characterized by Weiss *et al*. [18]. Raw RNA-seq data were retrieved from the National Center for Biotechnology Information (NCBI) Sequence Read Archive (SRA) under BioProject PRJNA952510. The original study generated and validated the ECFC cohort and analyzed a subset of samples (*n* = 35) to investigate long noncoding RNA expression in relation to maternal gestational weight gain [18]. In contrast, the present study utilized the complete cohort (*n* = 60) to examine protein-coding transcriptional responses across lean, overweight, and gestational diabetes pregnancies. Analyses were further stratified by fetal sex and integrated with regulatory-network inference approaches to identify candidate transcriptional mechanisms associated with sex-dependent ECFC responses to maternal metabolic dysfunction.

ECFC identity was established in the original study through expression of endothelial markers [CD31 and von Willebrand factor (vWF)] and the absence of mesenchymal markers, including CD90 and α-smooth muscle actin (SMA) [7]. The original dataset was also generated from high-quality RNA preparations [RNA integrity number (RIN) > 7.0] suitable for transcriptomic analysis [18]. To complement these experimental validations, we performed an independent transcript-level assessment of cellular identity using canonical hematopoietic markers, including PTPRC (CD45) and CD14, as negative controls (Fig. 1). Expression profiles were examined across all 60 samples prior to cohort stratification to identify evidence of potential hematopoietic contamination and to assess sample homogeneity. This additional bioinformatic verification step provided independent support that the analyzed transcriptomes were derived predominantly from endothelial progenitor cells, thereby increasing confidence that downstream transcriptional patterns reflected ECFC-associated biological variation rather than major differences in cellular composition.

**Fig. 1.**
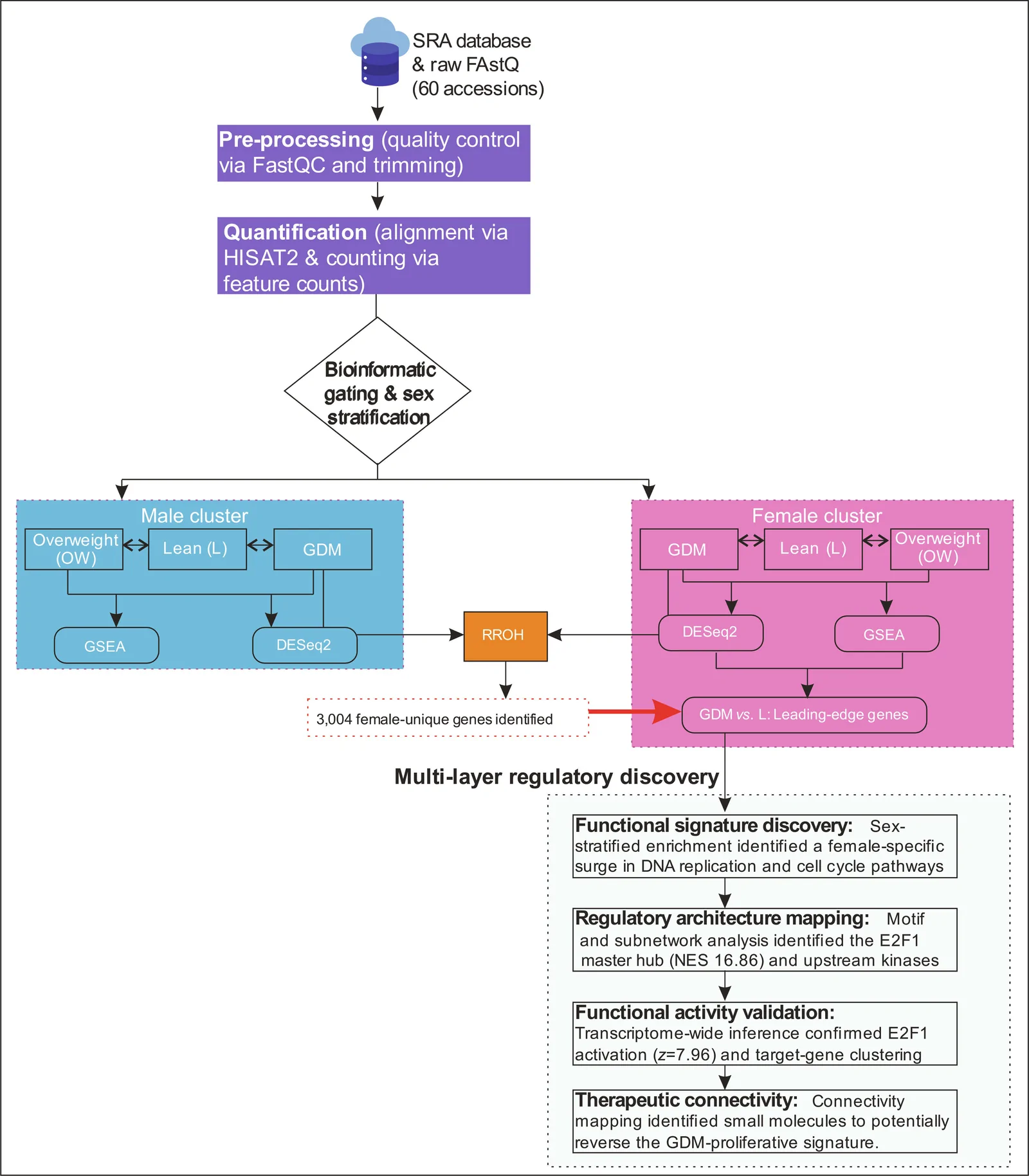
Integrated sex-stratified analytical workflow. Raw RNA-seq data from 60 ECFC samples were processed through quality control, alignment, quantification, and bioinformatic cell-identity verification prior to sex stratification. Differential-expression (DESeq2) and gene-set enrichment analyses (GSEA) were performed independently within male and female cohorts across lean, overweight, and GDM groups. RRHO analysis of the GDM-versus-lean comparison identified a female-specific transcriptional signature comprising 3,004 nominally associated transcripts. Leading-edge genes derived from enriched female pathways were subsequently subjected to pathway, transcription factor, kinase, and regulatory activity analyses using WebGestalt, GSVA, iRegulon, X2K, and decoupleR. Candidate pharmacological modulators associated with the female GDM transcriptional signature were identified using L1000FWD connectivity mapping.

### Bioinformatics, quality control, and transcriptome alignment

Raw FASTQ files underwent initial quality assessment using FastQC (v0.11.9) [19]. Adapter sequences and low-quality bases were removed using Trimmomatic (v0.39) [20], with a sliding-window trimming approach (4:20). Reads with Phred quality scores below 20 or lengths shorter than 50 base pairs were excluded from downstream analyses. Filtered paired-end reads were aligned to the human reference genome (GRCh38/hg38) using the splice-aware aligner HISAT2 (v2.2.1) [21]. Alignment files were converted to BAM format and coordinate-sorted using SAMtools (v1.13) [22]. Gene-level read counts were generated with featureCounts from the Subread package (v2.0.1) [23] using the GENCODE v44 primary genome annotation. Consistent with the default behavior of this annotation, quantification was performed across all annotated gene biotypes (protein-coding genes, long noncoding RNAs, small noncoding RNAs, pseudogenes, and immunoglobulin/T cell receptor gene segments; approximately 62,000 genes in GENCODE v44 in total), rather than being restricted to protein-coding genes alone (~19,400 genes in GENCODE v44). Only uniquely mapped reads were retained for expression quantification. Following count generation, genes with nonzero expression across the dataset were retained for downstream differential-expression and regulatory-network analyses; this expression-based filtering, rather than biotype restriction, accounts for the gene totals reported in subsequent sections

### Statistical framework and differential expression

Differential expression analyses were performed in R (v4.3.1) using DESeq2 (v1.50.2) [24]. To assess global transcriptomic structure, principal components analysis (PCA) was conducted on variance-stabilized expression values generated using the variance stabilizing transformation (VST) implemented in DESeq2 (Fig. 2). Batch-level metadata (e.g., sequencing run and processing date) were not available in the public deposition and could not be directly modeled; PCA did not reveal clustering patterns suggestive of strong batch effects, although this cannot be fully excluded given the absence of batch annotations. Differential expression was evaluated using a negative binomial generalized linear model (GLM) framework, with maternal metabolic status and fetal sex incorporated as experimental variables. For sex-stratified analyses, male and female samples were analyzed independently, resulting in balanced comparisons across lean, overweight, and gestational diabetes groups (*n* = 10 per group). Pairwise contrasts were performed for GDM versus Lean, GDM versus Overweight, and Overweight versus Lean using the Wald test following estimation of size factors and gene-wise dispersion parameters. Genes were considered differentially expressed if they satisfied a nominal *P* value < 0.05 and an absolute log₂ fold-change ≥ 0.58 (1.5-fold change). As the primary objective was to identify coordinated transcriptional programs for downstream pathway and regulatory-network analyses, nominal significance thresholds were retained for discovery analyses, while effect-size filtering was applied to reduce inclusion of minimal-expression changes. Standard DESeq2 quality-control procedures, including Cook’s distance-based evaluation of influential observations, were applied throughout the analysis.

**Fig. 2.**
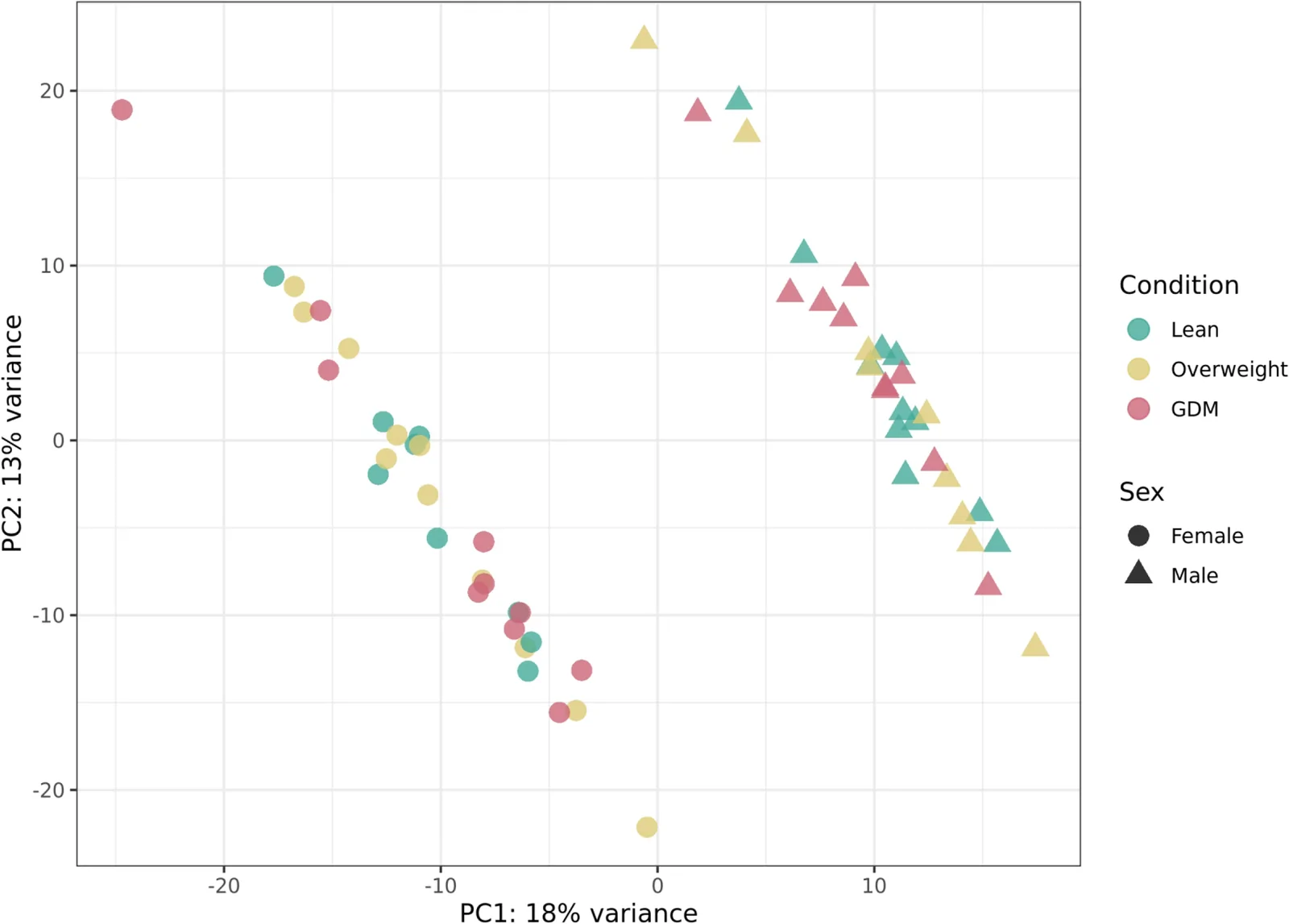
Fetal sex is the dominant axis of transcriptomic variance among neonatal ECFCs. PCA of VST counts for all 60 samples, restricted to the top 500 most variable genes (DESeq2 default). PC1 (18% variance) and PC2 (13% variance) represent the primary axes of transcriptomic diversity. Samples are colored by maternal metabolic status (Lean, Overweight, GDM) and shaped by neonatal sex (Female: circle, Male: triangle). Female and male samples separated almost entirely along PC1 (PERMANOVA on PC1–PC2, 999 permutations: *R* ^2^ = 0.556, *P* = 0.001; 59 of 60 samples correctly classified by sex based on PC1 sign), establishing sex as the dominant source of transcriptomic variance in this cohort prior to condition-based stratification.

### RRHO analysis

To compare transcriptomic responses between female and male ECFC cohorts, RRHO analysis was performed using the RRHO R/Bioconductor package [25,26]. RRHO is a threshold-free approach that evaluates overlap between 2 ranked gene lists across all possible rank combinations. For each sex-specific comparison, genes were ranked according to the direction and statistical significance of differential expression. Only genes detected in both female and male datasets were retained for analysis. RRHO was performed using a step size of 200 and the alternative = “enrichment” setting. Statistical significance was visualized as heatmaps of hypergeometric enrichment scores, with gene ranks from the female and male comparisons represented along the *x* and *y* axes, respectively.

### Sexual dimorphism validation and sex-covariate modeling

To evaluate the extent of sex-specific transcriptional responses associated with GDM, 2 complementary analyses were performed. First, differential expression results from the male and female GDM-versus-Lean comparisons were compared using Pearson and Spearman correlation analyses based on log_2_ fold-change estimates for genes detected in both datasets. Second, an alternative differential expression model was fitted to the complete cohort using sex as a covariate rather than a stratification variable (design: ~ sex + condition). Differential expression results obtained from the covariate-adjusted model were compared with those generated from the sex-stratified analyses to assess the influence of analytical strategy on the identification of GDM-associated transcriptional programs.

### Functional enrichment analysis and gene set variation analysis

Functional enrichment analysis was performed using the WebGestalt platform (v2019) [27]. Gene set enrichment analysis (GSEA) was conducted using ranked differential expression results to evaluate pathway-level changes across the transcriptome. Reactome was used as the primary pathway reference database. Pathways containing between 15 and 500 genes were retained for analysis. Statistical significance was assessed using the normalized enrichment score (NES) and a false discovery rate (FDR) threshold of 0.05 following Benjamini–Hochberg correction [28]. To evaluate pathway activity at the individual-sample level, gene set variation analysis (GSVA) was performed using the GSVA R/Bioconductor package [18]. Variance-stabilized expression values generated using DESeq2 served as input. Ensembl identifiers were converted to official gene symbols using the clusterProfiler (v4) [29] and org.Hs.eg.db annotation resources. When multiple Ensembl identifiers mapped to the same gene symbol, expression values were aggregated using the arithmetic mean. GSVA enrichment scores were calculated using the MSigDB hallmark gene-set collection (v2023.2) [30,31] with the Gaussian kernel cumulative distribution function (kcdf = “Gaussian”). GSVA pathway activity scores for the female-specific comparisons (GDM versus Lean, GDM versus Overweight, Overweight versus Lean) were analyzed using a linear model fit with limma (v3.66.0) [32], with maternal metabolic group as the independent variable and pairwise contrasts extracted for each comparison of interest. Differential pathway activity was assessed using a moderated *t* test with Benjamini–Hochberg correction for multiple testing, consistent with the approach applied to the sex × GDM interaction GSVA model described below. The Wilcoxon rank-sum test reported in Fig. S1 was used as a separate, nonparametric check on group differences within specific pathways of interest and is distinct from this primary limma-based analysis.

### Sex × GDM interaction testing

To directly test whether the transcriptional effect of GDM differed significantly between sexes, we fit a factorial model to the full 60-sample cohort using DESeq2 (v1.50.2) in R (v4.5.2) [24], with design formula ~sex + condition + sex:condition. The sex × condition interaction coefficients were extracted for GDM-versus-Lean and Overweight-versus-Lean. For pathway-level interaction testing, genes were ranked by the interaction Wald statistic and analyzed using fgsea (v1.36.2) [33] against the MSigDB hallmark collection (50 gene sets), retrieved via the msigdbr package (v26.1.1) [34]. As a complementary approach, GSVA pathway activity scores were analyzed using a factorial model with limma (v3.66.0) [32], and the corresponding sex × condition interaction coefficient was extracted directly from the model. This analysis was performed using a more recent R/Bioconductor environment (R v4.5.2) than the primary differential-expression analysis (R v4.3.1) [24]; core DESeq2 methodology for negative-binomial GLM fitting and Wald testing was unchanged between versions.

### Transcription factor inference and network topology analyses

To identify candidate transcription factors associated with the female-specific GDM transcriptional signature, transcription factor motif enrichment analysis was performed using the iRegulon plugin (v1.3) within Cytoscape (v3.10.1) [35,36]. Leading-edge genes derived from the enrichment analyses were evaluated against a reference collection of 9,713 position weight matrices (PWMs) and 1,120 ENCODE-derived chromatin immunoprecipitation sequencing (ChIP-seq) tracks [37]. Motif discovery was conducted using the genomic ranking database centered ±20 kb around transcription start sites. Candidate regulators were identified using an enrichment threshold of NES > 3.0, with an area under the cumulative recovery curve (AUC) threshold of 0.03 and a maximum motif-similarity FDR of 0.001. Potential upstream regulatory relationships were further explored using the Expression2Kinases (X2K) platform [38]. The 27 female-specific leading-edge genes identified by GSEA were used as the query set. Transcription factor enrichment was performed using ChEA3 [39], which evaluates overlap between query genes and transcription factor target-gene collections assembled from multiple experimental datasets. Protein–protein interaction networks were subsequently generated to identify intermediate interaction partners linking enriched transcription factors to upstream signaling components. Kinase enrichment analysis was then performed using KEA3 [40], and candidate kinases were ranked according to their mean rank across multiple interaction resources, including BioGRID, STRING, and Kinase Library.

### Transcription factor activity inference via decoupleR

Transcription factor activity analysis was performed using the decoupleR R/Bioconductor package [41–44]. This approach estimates transcription factor activity from the expression patterns of known target genes rather than relying solely on transcription factor expression levels. Regulatory interactions were obtained from the CollecTRI database [42], which provides curated transcription factor–target relationships. Activity scores were calculated using the univariate linear model (ULM) method implemented in decoupleR. In this framework, differential expression statistics were used together with known regulatory interactions to estimate the relative activity of individual transcription factors across the transcriptomic dataset. Prior to analysis, Ensembl identifiers were converted to official HUGO gene Nomenclature Committee (HGNC) gene symbols using the biomaRt package and the Ensembl BioMart database [45,46]. For visualization purposes, genes belonging to the CollecTRI-curated E2F1 target set were identified and highlighted on volcano plots generated using ggplot2 and ggrepel. Differential expression significance and effect size were represented by nominal *P* values and log₂ fold-change estimates, respectively.

### Comparison with independent datasets

To contextualize the present findings against independently published transcriptomic studies, 2 external datasets were identified through literature search: GSE296585 [47], comprising RNA-seq data from cord-blood-derived ECFCs from diabetic and healthy donors (cell type matching the present study; donor sex not recorded in the associated metadata), and GSE103552 [48], comprising sex-stratified RNA-seq data from feto-placental endothelial cells from GDM and control pregnancies. These datasets were not reanalyzed from raw data by the present authors; findings reported here reflect the results and conclusions published by the original authors, cited for comparison against the present study rather than as an independent replication using a shared analytical pipeline.

### Connectivity mapping and small-molecule reversal analysis

To identify compounds associated with transcriptional patterns similar or dissimilar to the female GDM expression signature, connectivity mapping was performed using the L1000FWD (L1000 Fireworks Display) platform [4]. L1000FWD compares an input gene-expression signature against a large reference collection of perturbation-induced transcriptional profiles generated through the LINCS L1000 project. The query signature consisted of the top 50 up-regulated and top 50 down-regulated genes identified in the female GDM differential expression analysis, based on statistical significance and effect size. For each perturbation, L1000FWD computes a connectivity score ranging from −1 to +1 that reflects the degree of similarity between the query signature and the reference expression profile. Negative connectivity scores indicate transcriptional patterns that oppose the input signature, whereas positive scores indicate transcriptional patterns with greater similarity to the observed expression changes. Compounds identified through connectivity mapping were subsequently grouped according to their reported mechanisms of action to facilitate biological interpretation and comparison with pathways highlighted by the upstream regulatory analyses.

## Results

### Construction and validation of the bioinformatic pipeline

Raw RNA-seq data retrieved from the SRA were first subjected to quality assessment using FastQC. Per-base sequence quality scores remained consistently above a Phred score of 36 across the read length, corresponding to a base-calling accuracy exceeding 99.97% (Fig. 1). A modest deviation in nucleotide composition was observed within the first ~14 bases of the reads, consistent with patterns commonly associated with random-hexamer priming during library preparation. Sequence quality remained uniform across flowcell tiles and samples. Filtered reads were aligned to the human reference genome (GRCh38/hg38) using HISAT2. The mean alignment rate across the cohort was approximately 98%, indicating successful mapping of sequencing reads to the reference genome. Duplicate reads were subsequently identified using Picard MarkDuplicates and flagged prior to quantification. The average duplication rate across samples was approximately 23%. Gene-level quantification was then performed using featureCounts on the filtered alignment files.

Transcript-level assessment of cellular identity was performed using established endothelial and hematopoietic marker genes. Expression of the hematopoietic markers *PTPRC* (CD45) and *CD14* was negligible or below detection across all 60 samples, whereas the endothelial markers *PECAM1* (CD31) and *VWF* displayed consistent expression throughout the cohort.

### PCA demonstrates strong baseline transcriptomic separation by fetal sex

PCA of the ECFC transcriptomes showed that PC1 and PC2 accounted for 18% and 13% of the total variance, respectively (Fig. 2). Samples separated pronouncedly by fetal sex along PC1, with female and male samples occupying almost entirely distinct regions of PCA space; a formal test of this separation [permutational multivariate analysis of variance (PERMANOVA) on joint PC1–PC2 coordinates, 999 permutations] confirmed that fetal sex explained over half of the variance captured by these 2 components (*R* ^2^ = 0.556, *P* = 0.001). Based on PC1 sign alone, 59 of 60 samples fell on the expected side for their sex, a post hoc descriptive observation rather than a cross-validated classification estimate. Within each sex, GDM-exposed ECFCs occupied a partially distinct distribution along PC2 relative to Lean and Overweight controls, although overlap remained between metabolic-status groups. To evaluate GDM-associated transcriptional changes across the complete dataset, differential expression analysis was initially performed without sex stratification using all 60 samples. This analysis identified no significantly differentially expressed genes between GDM and Lean groups following multiple-testing correction (adjusted *P* < 0.05). This strong baseline separation by sex, together with the absence of significant differential expression in the pooled analysis, motivated a sex-stratified analytical approach; whether the effect of GDM itself differs significantly between sexes is addressed directly below. Subsequent analyses were therefore conducted separately for male and female cohorts, a conclusion further supported by the male–female correlation and sex-covariate analyses presented in the Sex stratification reveals distinct female and male transcriptional responses to GDM section.

### Sex-stratified transcriptomic profiling and identification of metabolic signatures

Differential expression analysis was performed to compare the transcriptomic profiles of Lean, Overweight, and GDM ECFCs using a significance threshold of *P* < 0.05 and an absolute fold-change ≥1.5. Unsupervised hierarchical clustering reveals substantial heterogeneity within metabolic groups (Fig. 3). Nevertheless, sex-stratified analyses identified distinct sets of nominally associated genes associated with maternal metabolic status. Within the female cohort, comparison of Overweight and Lean samples identified increased expression of *FAP* (*fibroblast activation protein*), *SLITRK4*, and *CORIN,* while differential expression of *AKT3* and *CETP* characterized the male cohort. When GDM samples were compared directly with Overweight controls, additional sex-associated differences were observed. Female GDM ECFCs showed increased expression of genes including *HLA-C*, *TAGLN*, and *KIFC1*, whereas differential expression of *PCSK1*, *ITGA4*, and *HAPLN1* characterized the males, with increased expression across multiple male-group comparisons. Direct comparison of GDM and Lean samples further highlighted differences between male and female transcriptional responses to maternal metabolic dysfunction. These sex-specific expression patterns provided the basis for subsequent pathway and regulatory-network analyses.

**Fig. 3.**
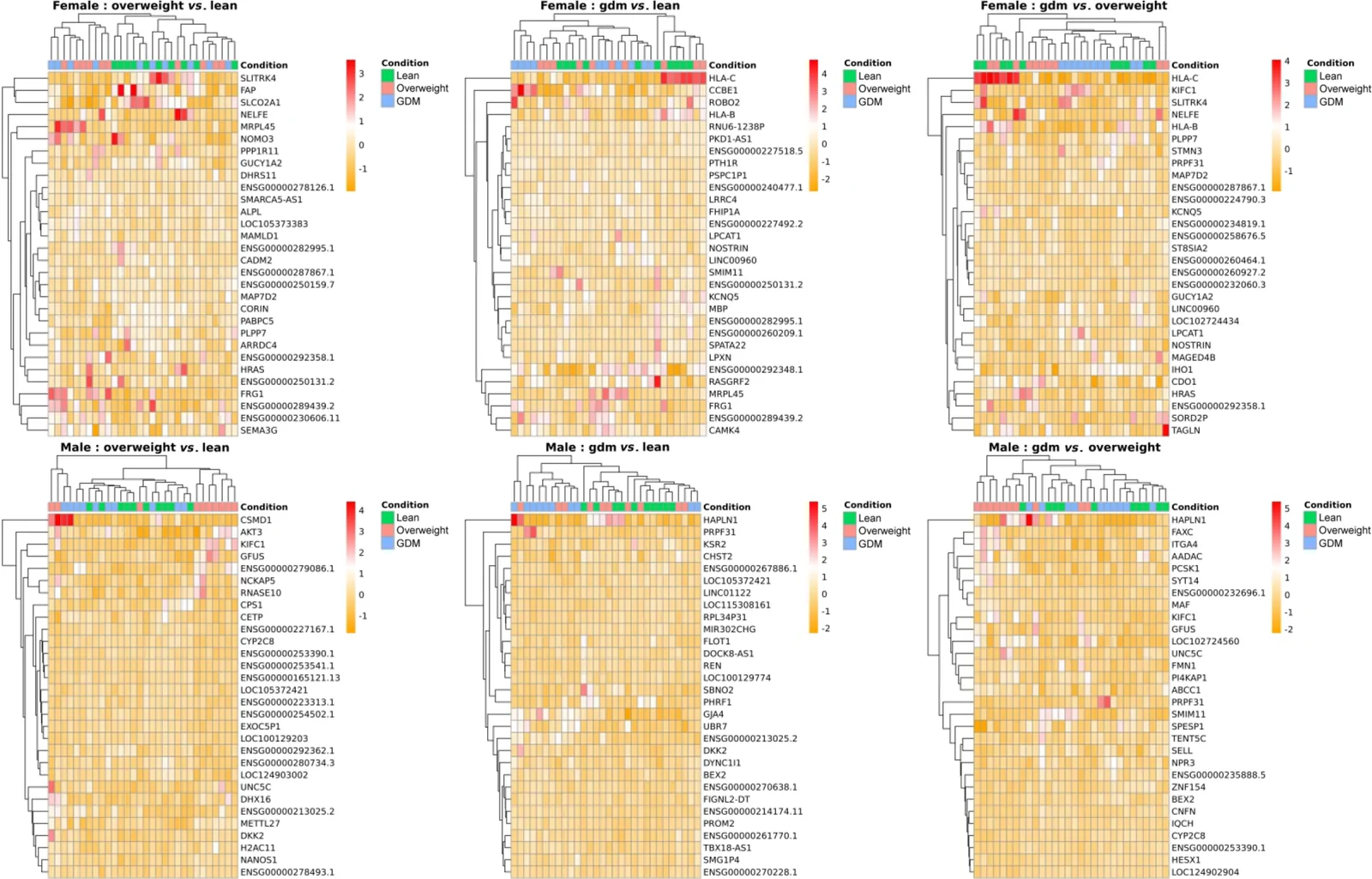
Sex-stratified transcriptomic profiling of ECFCs across maternal metabolic conditions. Heatmaps showing the top 30 top-ranked nominally associated genes identified in female (top row) and male (bottom row) cohorts. Comparisons include Overweight versus Lean, GDM versus Lean, and GDM versus Overweight. These genes were defined using a nominal *P* value < 0.05 and an absolute log_2_ fold-change ≥ 0.58. Rows represent individual genes (HGNC symbols), and columns represent individual samples. Colors indicate relative expression levels based on row-scaled *z*-scores, with higher expression shown in red and lower expression shown in orange. Dendrograms represent unsupervised hierarchical clustering of genes and samples based on expression similarity.

### Sex stratification reveals distinct female and male transcriptional responses to GDM

The sex-specific nature of the GDM-associated transcriptional response was evaluated using complementary analyses. Genome-wide comparison of differential expression results between male and female GDM-versus-Lean contrasts revealed minimal concordance between the sexes. Across 35,883 shared genes, fold-change estimates were uncorrelated (Pearson *r* = −0.01; Spearman *r* = −0.012), indicating that male and female ECFCs exhibited substantially different transcriptional responses to GDM exposure. To further assess the influence of analytical design, differential expression analysis was repeated using the complete cohort with sex included as a covariate rather than as a stratification variable (design = ~ sex + condition). Under this approach, no genes met the significance threshold following multiple-testing correction (adjusted *P* < 0.05). Similarly, genes comprising the E2F-associated cell-cycle signature identified in the female-specific analysis, including *E2F1*, *MCM2–7*, *CDC6*, and *CDC25A*, exhibited small effect sizes (|log₂FC| < 0.19) and nonsignificant adjusted *P* values (Table S1). Fold-change estimates derived from the covariate-adjusted model correlated similarly with those obtained from the male-only (*r* = 0.68) and female-only (*r* = 0.70) analyses. Together, these findings indicate that treating sex as a covariate did not recover the female-specific transcriptional patterns identified through sex-stratified analyses.

### Comparative functional enrichment and transcriptomic overlap mapping

Comparison of pathway enrichment profiles across the 3 metabolic contrasts revealed marked differences between female and male ECFCs (Fig. 4). In the Overweight-versus-Lean comparison, female ECFCs showed enrichment of fibroblast growth factor receptor (FGFR) signaling and gap junction assembly pathways, including contributions from *FGFR1* and *FGFR4* (FDR < 0.05). In contrast, male ECFCs exhibited significant negative enrichment of Cholesterol Biosynthesis, Extracellular Matrix Organization, and WNT-Dependent Signaling pathways. The GDM-versus-Lean comparison revealed additional differences between the sexes. In female ECFCs, positively enriched pathways included Activation of ATR in Response to Replication Stress, S Phase, and Mitotic Prometaphase. Negative enrichment was observed for Regulation of Complement Cascade, Interferon-Gamma Signaling, and Interleukin-10 Signaling pathways. No pathways reached statistical significance after multiple-testing correction (FDR < 0.05) in the corresponding male comparison. To distinguish transcriptional changes associated with GDM from those associated with overweight status, GDM samples were compared directly with Overweight controls. Female ECFCs again demonstrated enrichment of cell-cycle-associated pathways, including G1/S Transition. In contrast, male ECFCs showed positive enrichment of Cholesterol Biosynthesis and Viral mRNA Translation pathways. Notably, Cholesterol Biosynthesis was negatively enriched in the Overweight-versus-Lean comparison but positively enriched in the GDM-versus-Overweight comparison, indicating different pathway responses across the 2 metabolic transitions in the male cohort.

**Fig. 4.**
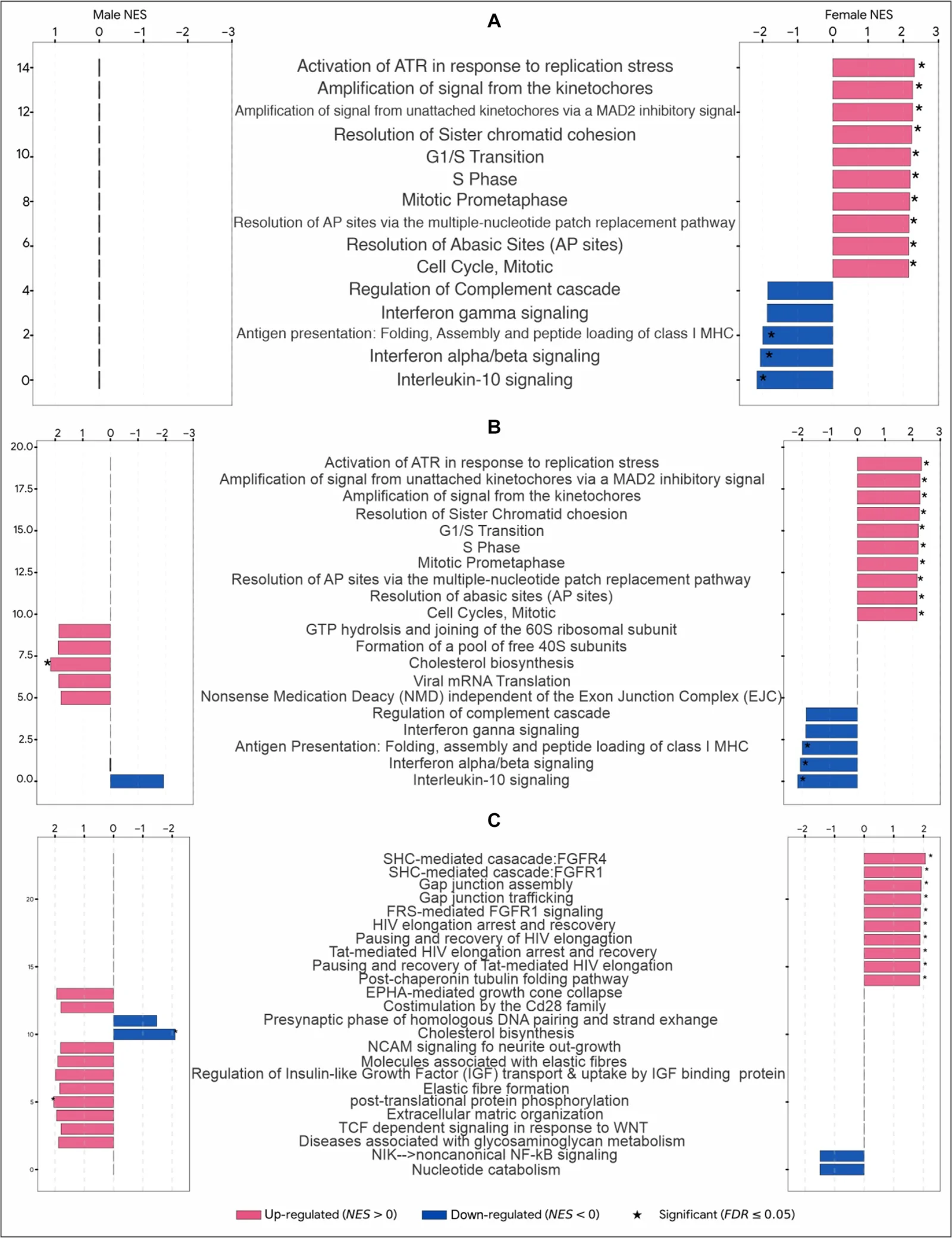
Comparative pathway enrichment across maternal metabolic conditions in female and male ECFCs. GSEA results are shown for 3 pairwise comparisons: (A) GDM versus Lean, (B) GDM versus Overweight, and (C) Overweight versus Lean. In each panel, the left axis and bars correspond to NES in the male cohort, and the right axis and bars correspond to NES values in the female cohort, with pathway names shown between the 2. Pink bars indicate up-regulated pathways (NES > 0), and blue bars indicate down-regulated pathways (NES < 0); asterisks denote statistical significance (FDR ≤ 0.05). Pathways were annotated using the Reactome database and analyzed through the WebGestalt platform. In the GDM-versus-Lean comparison (A), no pathways reached the enrichment threshold in male ECFCs, whereas female ECFCs showed significant enrichment of cell-cycle and DNA replication-associated pathways (e.g., Activation of ATR in Response to Replication Stress, G1/S Transition, S Phase, and Mitotic Prometaphase) alongside significant down-regulation of immune-signaling pathways (e.g., Interferon alpha/beta signaling and Interleukin-10 signaling). In contrast, both sexes showed pathway-level enrichment in the GDM-versus-Overweight (B) and Overweight-versus-Lean (C) comparisons, indicating that the absence of a male transcriptional response is specific to the GDM-versus-Lean contrast rather than a general feature of the male cohort.

### Integrative convergence and divergence analysis (RRHO)

Male and female GDM-associated transcriptional responses were compared using Venn analysis and RRHO. Venn analysis identified genes shared between the 2 responses, whereas RRHO was used to determine whether the overall transcriptional changes observed in females were also present in males. As reported above, no genes reached FDR-corrected significance in either sex-stratified GDM-versus-lean contrasts; the gene counts reported below therefore reflect nominally associated transcripts used for exploratory overlap and network analyses, rather than FDR-confirmed differential expression. Greater overlap was observed among down-regulated genes than among up-regulated genes. A total of 828 nominally associated down-regulated transcripts were shared between the sexes, whereas 3,004 of such transcripts were unique to the female cohort. Among up-regulated genes, only 116 nominally associated transcripts were common to both male and female (Fig. 5A). Genome-wide concordance was further assessed using threshold-free RRHO analysis. The resulting heatmap revealed a significant enrichment hotspot in the upper-right quadrant (−log₁₀ *P* > 35), corresponding to genes that were down-regulated in both female and male GDM comparisons (Fig. 5B). In contrast, the lower-left quadrant, representing genes up-regulated in both sexes, showed comparatively limited enrichment, indicating less concordance among up-regulated transcriptional responses. To examine biological processes associated with sex-specific transcriptional changes, functional enrichment analysis was performed on the female-specific down-regulated gene set. Enriched pathways included transforming growth factor-β (TGF-β) signaling, extracellular matrix organization, and Janus kinase (JAK)–signal transducer and activator of transcription 3 (STAT3) signaling. Pathway-level analyses additionally identified enrichment of DNA replication- and cell-cycle-associated pathways within the female GDM response. In comparison, male GDM-associated transcriptional changes were characterized primarily by enrichment of metabolic pathways. Given the larger female-specific transcriptional signature and the associated pathway enrichment, subsequent regulatory-network analyses focused on the female GDM cohort to identify candidate transcriptional regulators associated with these expression patterns.

**Fig. 5.**
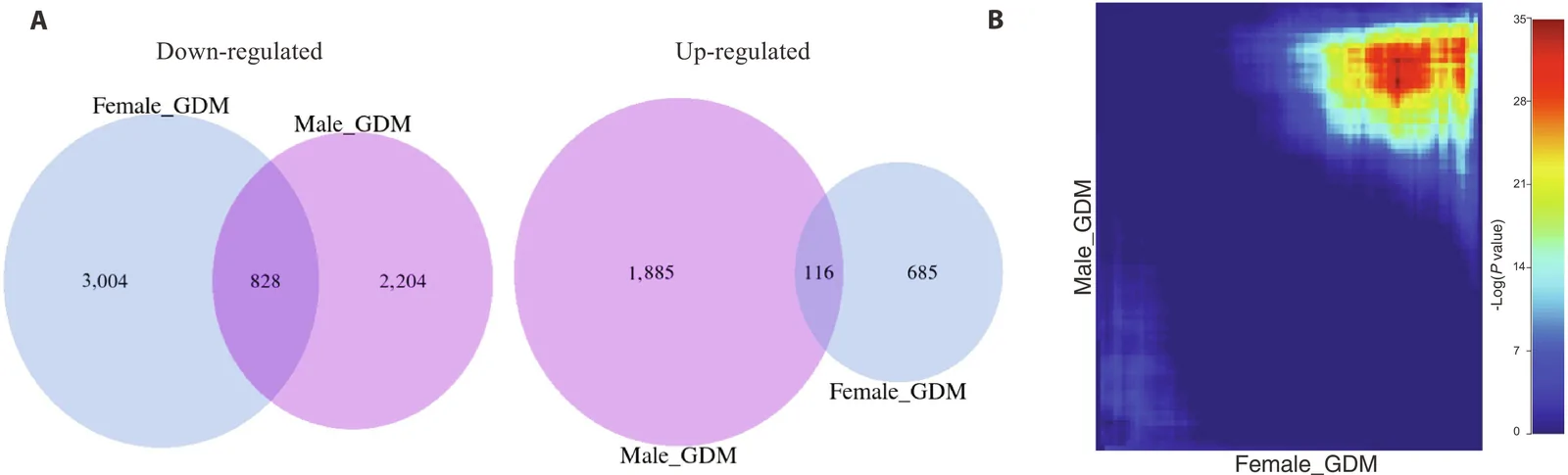
RRHO analysis of male and female GDM-associated transcriptional responses. (A) Venn diagrams showing overlap between nominally associated genes identified in female and male GDM-versus-Lean comparisons. Down-regulated genes exhibited greater overlap between the sexes than up-regulated genes, while a larger number of sex-specific genes were identified in the female cohort. Numbers indicate gene counts within each category. (B) RRHO significance map comparing ranked gene-expression signatures from female (*x* axis) and male (*y* axis) GDM-versus-Lean analyses. Color intensity represents the statistical significance of overlap between ranked gene lists, expressed as −log_10_(*P* value) from the hypergeometric test. The upper-right quadrant contains genes exhibiting concordant regulation in both sexes, whereas other regions represent gene sets with differing rank distributions between the 2 comparisons. The observed enrichment pattern indicates greater concordance among down-regulated genes than among up-regulated genes.

#### GSVA reveals female-specific pathway polarization

Previous analyses revealed limited concordance between male and female GDM-associated transcriptional responses, together with a substantially larger female-specific transcriptional signature. Consistent with these findings, pathway enrichment analyses identified significant pathway-level changes in the female cohort, whereas comparatively few pathways reached significance in the corresponding male analyses. To further characterize the female GDM signature, GSVA was performed. Whereas GSEA identified pathways enriched at the group level, GSVA was used to determine whether these pathway-level changes were consistently represented across individual samples (Fig. 6). Female GDM ECFCs exhibited higher enrichment scores for several cell-cycle-associated pathways, including E2F Targets, G2M Checkpoint, Mitotic Spindle, and Hedgehog Signaling, although these group-level trends did not reach FDR-corrected significance (Fig. 6). In contrast, lower enrichment scores were observed for IL6-JAK-STAT3 Signaling, Angiogenesis, Inflammatory Response, Epithelial-Mesenchymal Transition (EMT), and Bile Acid Metabolism pathways. Similar enrichment patterns were observed across individual female GDM samples and were consistent with the pathway-level trends identified by GSEA. Thus, the pathway signatures identified at the cohort level were also evident at the individual-sample level. To determine whether this pathway-level signature was similarly present in male ECFCs, the identical GSVA-based limma analysis (GDM versus Lean, all 50 MSigDB Hallmark gene sets) was performed in the male cohort. No pathways reached nominal (*P* < 0.05) or FDR-corrected significance in male ECFCs. Notably, the 2 pathways most directly relevant to the female-specific signature, E2F Targets and G2M Checkpoint, trended in the opposite direction in males (log_2_FC = −0.058 and −0.055, respectively) relative to the positive, nonsignificant trend observed in females (log_2_FC = +0.243, *P* = 0.106, FDR = 0.990 and log_2_FC = +0.233, *P* = 0.088, FDR = 0.990, respectively; Fig. S1), further supporting the female-specific nature of the proliferative response to GDM.

**Fig. 6.**
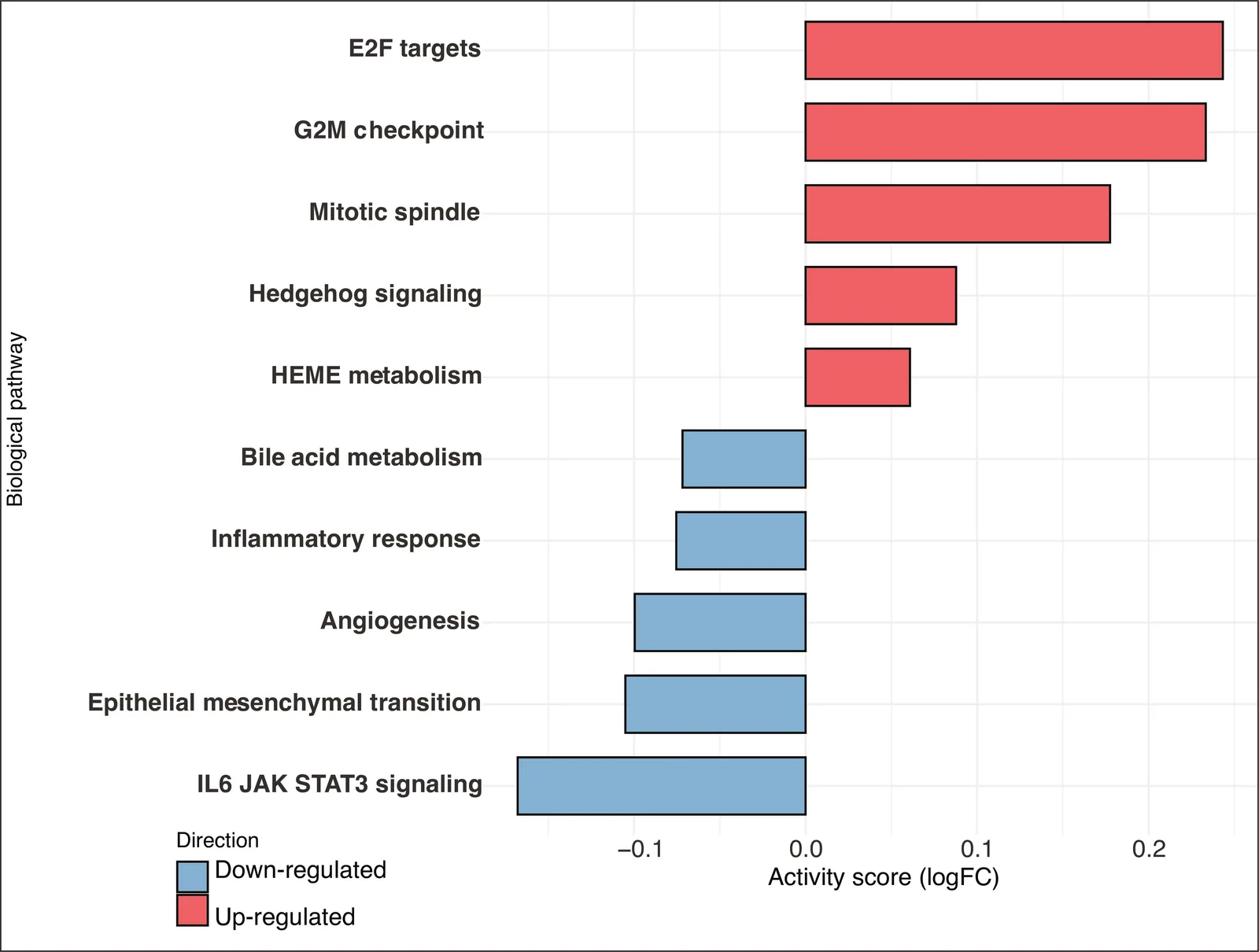
GSVA-based pathway activity profile in female ECFCs, GDM versus Lean. Bar chart showing differential pathway activity scores (logFC) derived from GSVA using the MSigDB hallmark gene-set collection. Positive values (red bars) indicate higher pathway activity scores in GDM samples relative to Lean controls, whereas negative values (blue bars) indicate lower activity scores. Positively enriched pathways included E2F Targets, G2M Checkpoint, Mitotic Spindle, Hedgehog Signaling, and HEME Metabolism. Pathways with lower activity scores included IL6-JAK-STAT3 Signaling, Angiogenesis, Inflammatory Response, Epithelial-Mesenchymal Transition (EMT), and Bile Acid Metabolism. Pathway activity scores were calculated from individual-sample transcriptomic profiles and are shown as GSVA-derived logFC values.

#### Transcription factor enrichment analysis prioritizes E2F-associated regulatory programs in female GDM ECFCs

To identify candidate transcription factors associated with the female-specific GDM transcriptional signature, motif enrichment analysis was performed using iRegulon on the 27 female leading-edge genes identified by GSEA. Multiple members of the E2F transcription factor family were among the highest-ranked enriched regulators (Table 1). The strongest enrichment was observed for the E2F1/E2F4 cluster (mean NES = 16.42), followed by E2F7/E2F8 (mean NES = 16.14) and E2F3/E2F5 (mean NES = 15.30). Predicted target genes associated with these transcription factors included several components of DNA replication and cell-cycle regulation, including *MCM2–7*, *CDC6*, *CDC25A*, and *CHEK1*. Considerable overlaps were observed among the target sets identified for the different E2F family clusters, with genes involved in DNA replication licensing and cell-cycle progression represented repeatedly across enriched regulons (Table 1). Comparable enrichment of E2F-associated transcription factor clusters was not observed in the corresponding male analysis. Together, these findings identify multiple members of the E2F family as highly ranked candidate regulators associated with the female GDM transcriptional signature.

**Table 1.** Enrichment of transcription factor binding motifs in female GDM leading-edge genes

Regulatory cluster	Primary candidate	Mean NES	Total unique targets	Representative target genes
E2F activation hub	E2F1/E2F4	16.42	18	*MCM2–7*, *CDC6*, *CDC25A*, *CHEK1*
S-phase repressors	E2F7/E2F8	16.14	17	*MCM6*, *CDC6*, *MCM5*, *CDC25A*
Late-cycle regulators	E2F3/E2F5	15.30	13	*MCM3*, *MCM5*, *MCM4*, *CDC25A*

#### Network and activity inference analyses prioritize an E2F-associated regulatory framework

To further investigate candidate upstream regulators associated with the female GDM transcriptional signature, leading-edge genes were analyzed using the X2K framework, which links transcriptional signatures to candidate transcription factors, protein-interaction networks, and upstream kinases (Fig. 7). Kinase enrichment analysis identified *CHEK2*, *CDK2*, and *BUB1B* among the highest-ranked kinases across multiple reference libraries (Fig. 7A). In parallel, transcription factor enrichment using ChEA3, which prioritizes regulators based on experimentally derived transcription factor–target relationships, highlighted *E2F7*, *FOXM1*, and *E2F1* among the highest-ranked candidate regulators supported by ChIP-seq and coexpression datasets (Fig. 7B). Integration of kinase and transcription factor enrichment results generated a regulatory network connecting kinase-associated signaling components, including *ATR*, *ATM*, and *CDK1/2*, with transcription factors such as *E2F1*, *SP1*, and *MYBL2* (Fig. 7C). Several genes previously identified within DNA replication and cell-cycle-associated pathways, including *MCM2*, *MCM6*, *CDC6*, and *CDC25A*, were incorporated within this inferred network structure. To further evaluate transcription factor activity, decoupleR analysis was performed using the CollecTRI regulatory database. Unlike motif enrichment approaches, decoupleR estimates transcription factor activity from the expression patterns of known downstream target genes. Consistent with the transcription factor enrichment analyses, multiple genes belonging to the E2F-associated target network exhibited increased expression in the female GDM comparison. In contrast, transcript levels of *E2F1*, *MYBL2*, and *FOXM1* showed relatively small fold changes (|log₂FC| < 0.25; *P* > 0.05) (Fig. 7D). Collectively, the iRegulon, ChEA3, X2K, and decoupleR analyses independently prioritized members of the E2F transcription factor family together with multiple cell-cycle-associated kinases, including *CDK1*, *CDK2*, *ATR*, and *ATM*, as candidate regulators associated with the female GDM transcriptional signature.

**Fig. 7.**
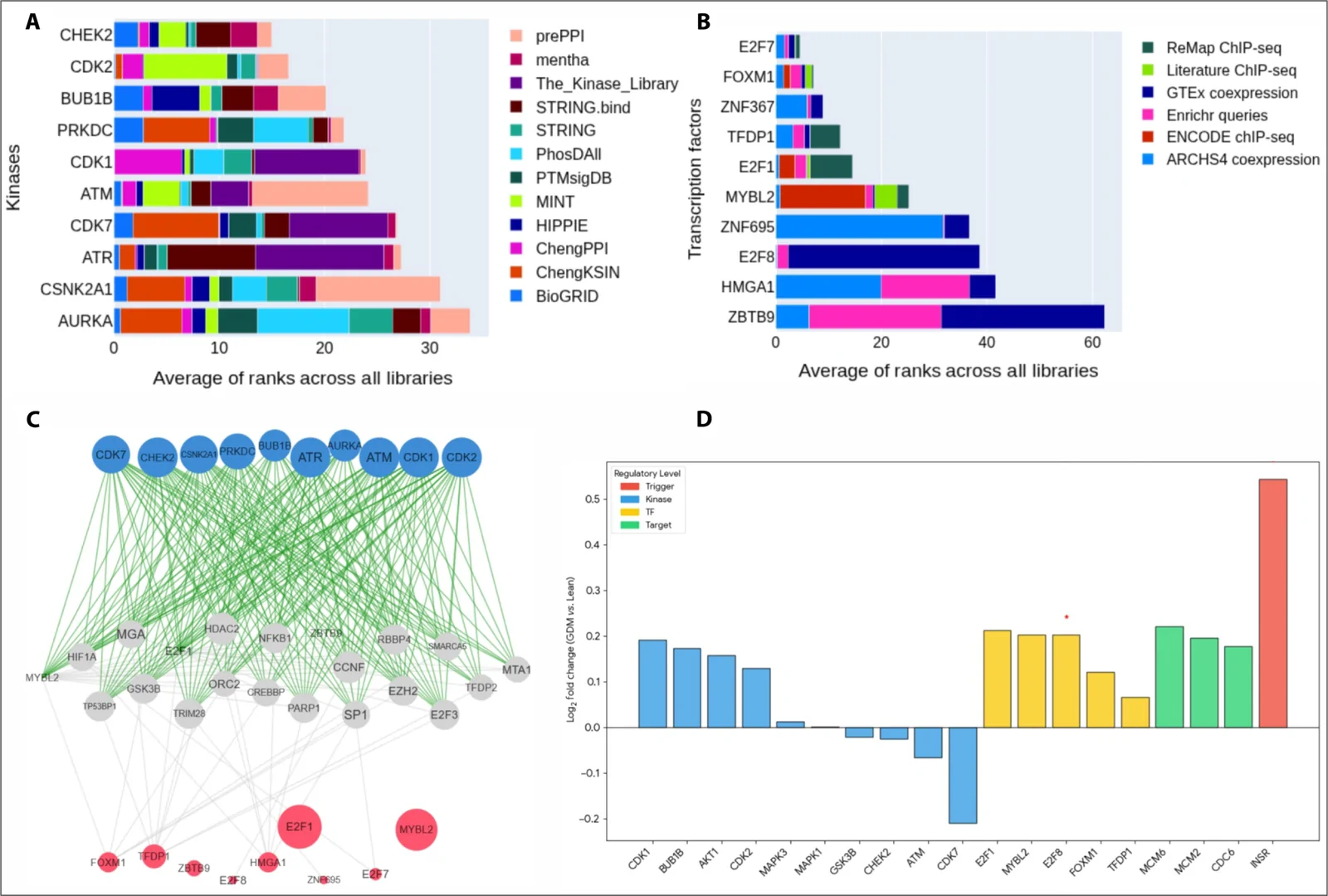
Network and activity inference analyses associated with the female GDM transcriptional signature. (A) Kinase enrichment analysis (KEA3) performed on female GDM leading-edge genes. Bar plots show the highest-ranked kinases identified across multiple reference libraries, including BioGRID, STRING, and Kinase Library. Kinases are ranked according to their mean enrichment score across datasets. (B) Transcription factor enrichment analysis (ChEA3) of female GDM leading-edge genes. Candidate transcription factors were prioritized using integrated ChIP-seq, coexpression, and regulatory datasets. The highest-ranked transcription factors identified across datasets are shown. (C) Integrated X2K regulatory network linking candidate kinases, intermediate protein–protein interaction partners, transcription factors, and downstream target genes. Blue nodes represent kinases, red nodes represent transcription factors, and gray nodes represent intermediate interacting proteins. Edges represent protein–protein or regulatory interactions inferred by the X2K framework. (D) Expression profiles of selected signaling components, transcription factors, and downstream target genes identified through the integrated network analyses. Differential expression is shown for genes including *INSR*, *CDK1*, *CDK2*, *BUB1B*, *E2F1*, *MYBL2*, *MCM2*, *MCM6*, *CDC6*, and *CDC25A* in the female GDM-versus-Lean comparison. Asterisks indicate nominally significant differential expression (*P* < 0.05); none of these genes reached significance after FDR correction.

### Functional inference of transcription factor activity

To further evaluate transcription factor activity associated with the female GDM transcriptional signature, we performed activity inference using the decoupleR framework and the CollecTRI regulatory network (Table 2). Unlike motif enrichment approaches, decoupleR estimates transcription factor activity from the coordinated expression patterns of known downstream target genes. Activity scores were calculated using the ULM algorithm. Several members of the E2F transcription factor family were among the highest-ranked regulators inferred from the female GDM comparison. *E2F1* exhibited the highest activity score (*z* = 7.96, *P* = 1.77 × 10^−15^), followed by *E2F4* (*z* = 7.20), *E2F3* (*z* = 5.98), and *E2F2* (*z* = 5.70) (Table 2). Increased activity scores were also observed for *MYC* (*z* = 5.63), which ranked among the highest positively scored regulators in the analysis. In contrast, several transcription factors associated with immune and vascular regulatory processes exhibited negative activity scores. These included *IRF1* (*z* = −5.67, *P* = 1.42 × 10^−8^), *FOXF1* (*z* = −5.20), *HIVEP2* (*z* = −4.81), *REL* (*z* = −4.46), and *STAT1* (*z* = −4.21) (Table 2). Notably, the elevated activity scores inferred for *E2F1* and related E2F-family members occurred despite relatively stable transcript abundance of *E2F1* itself in the differential-expression analysis. In contrast, multiple genes belonging to the inferred E2F-associated target network, including *MCM2*, *MCM6*, *CDC6*, and *CDC25A*, exhibited increased expression in female GDM ECFCs (Fig. 7D). These activity patterns were consistent with the pathway-level analyses described above, which identified enrichment of cell-cycle-associated pathways together with reduced enrichment of multiple immune- and signaling-related pathways. Collectively, the decoupleR analysis prioritized members of the E2F family among the highest-scoring candidate regulators associated with the female GDM transcriptional signature.

**Table 2.** Top 10 inferred transcription factor activities in female GDM. ↑ means up-regulated, and ↓ means down-regulated.

Rank	TF	Activity score (z)	*P* value	Activity status	Primary biological function
1	E2F1	7.96	1.77e^−15^	**↑**	DNA replication and S-phase entry
2	E2F4	7.20	6.40e^−13^	**↑**	G1/S transition and cell cycle control
3	E2F3	5.98	2.28e^−9^	**↑**	Regulation of cell proliferation
4	E2F2	5.70	1.23e^−8^	**↑**	Orchestration of cell cycle progression
5	IRF1	−5.67	1.42e^−8^	**↓**	Innate immune response and IFN signaling
6	MYC	5.63	1.82e^−8^	**↑**	Metabolic reprogramming and biosynthesis
7	FOXF1	−5.20	1.97e^−7^	**↓**	Angiogenesis and vascular development
8	HIVEP2	−4.81	1.97e^−7^	**↓**	Inflammatory homeostasis
9	REL	−4.46	8.42e^−6^	**↓**	NF-κꞵ-mediated immune survival
10	STAT1	−4.21	2.61e^−5^	**↓**	Type-I/II interferon alarm response

### Visualization of E2F-associated target-gene expression patterns

To visualize the distribution of E2F-associated target genes within the female GDM transcriptional response, genes belonging to the CollecTRI-curated E2F regulatory network were highlighted on the global female GDM-versus-Lean volcano plot (Fig. 8). E2F-associated target genes were distributed across both up-regulated and down-regulated regions of the transcriptome. Among the most strongly up-regulated targets was *FBN2* (log₂FC > 2.0), whereas *ANGPT2* and *DGKI* exhibited substantial negative fold changes (log₂FC < −1.0). Additional E2F-associated targets, including *FHIT*, were also distributed across the volcano plot. The presence of E2F-associated target genes among both positively and negatively regulated transcripts indicates that genes linked to the inferred E2F regulatory network were not restricted to a single direction of expression change. Instead, these genes were represented across multiple regions of the female GDM transcriptional landscape (Fig. 8). This distribution provides a visual representation of the broad transcriptional footprint associated with the E2F-linked regulatory program identified through the preceding network and activity-inference analyses.

**Fig. 8.**
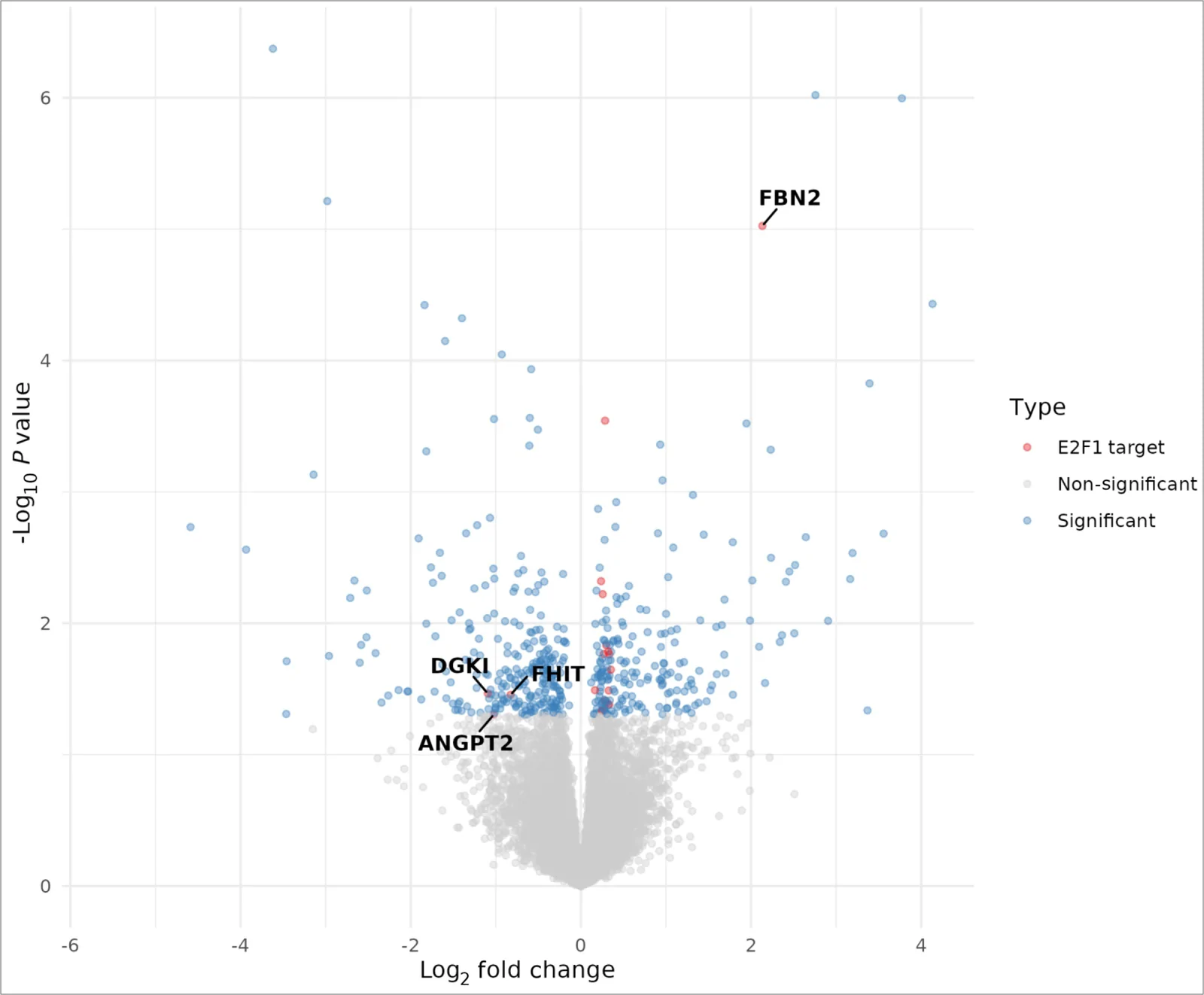
Distribution of E2F-associated target genes within the female GDM transcriptional landscape. Volcano plot showing differential gene expression in female GDM ECFCs relative to Lean controls. Each point represents a single gene and is plotted according to its log_2_ fold change (*x* axis) and statistical significance (−log_10_ *P* value; *y* axis). Blue points represent genes meeting the differential-expression threshold, whereas gray points represent genes that did not meet the significance criteria. Red points highlight genes belonging to the CollecTRI-curated E2F regulatory network. Representative E2F-associated target genes, including *FBN2*, *ANGPT2*, *FHIT*, and *DGKI*, are labeled. The highlighted genes are distributed across both positively and negatively regulated regions of the transcriptome.

### A sex × GDM interaction is detectable at the pathway but not individual-gene level

To evaluate whether the transcriptional response to GDM differed significantly between sexes, we tested the sex × GDM interaction directly using 2 complementary approaches. At the individual-gene level, no genes reached FDR-corrected significance for the interaction term (582 nominal, 0 FDR-significant, of 41,780 genes tested), including each of the 10 core E2F1-regulon genes examined previously (interaction *P* = 0.136 to 0.639; all FDR = 1.000; Table 3). In contrast, GSEA performed on genes ranked by this interaction statistic identified HALLMARK_E2F_TARGETS (NES = 2.58, FDR = 1.2 × 10^−21^) and HALLMARK_G2M_CHECKPOINT (NES = 2.44, FDR = 7.1 × 10^−18^) as the 2 most significant sex-differential pathways genome-wide, out of 50 Hallmark gene sets tested, with 12 pathways reaching significance overall. In addition to these cell-cycle pathways, the interaction-ranked GSEA identified several immune- and inflammation-related pathways with significant negative interaction enrichment, including HALLMARK_IL6_JAK_STAT3_signaling (NES = 1.78, FDR = 1.4 × 10^−3^), HALLMARK_INTERFERON_GAMMA_RESPONSE (NES = 1.58, FDR = 1.5 × 10^−3^), HALLMARK_INFLAMMATORY_RESPONSE (NES = −1.50, FDR = 5.4 × 10^−3^), and HALLMARK_ALLOGRAFT_REJECTION (NES = 1.67, FDR = 3.6 × 10^−4^; full results available in the deposited analysis repository, see Data Availability). This indicates that the sex × GDM interaction is not restricted to cell-cycle programs: The suppression of immune and reparative signaling previously described in the female-specific signature is itself significantly sex-differential. A factorial limma model applied directly to GSVA pathway scores did not independently reproduce this result for either pathway (HALLMARK_E2F_TARGETS: *P* = 0.189, FDR = 0.984; HALLMARK_G2M_CHECKPOINT: *P* = 0.157, FDR = 0.984; Table 3), although the direction of the interaction effect was consistent with the GSEA result in both cases. This discrepancy likely reflects differences in statistical power between approaches: GSEA leverages the coordinated interaction statistics of all genes within a pathway (~200 genes) against a genome-wide background, whereas the GSVA-based test first collapses each pathway into a single per-sample score, reducing sensitivity to distributed, individually modest gene-level effects at this sample size (*n* = 10 per group).

**Table 3.** Sex × GDM interaction testing across 3 statistical approaches. All 10 E2F1-regulon genes are shown to report the full gene set tested; none reached FDR significance for the sex × GDM interaction. Pathway-level results are shown for both GSEA and GSVA + limma to illustrate the method-dependent discordance discussed in the main text. Direction of effect is positive across all rows.

Level	Method	Target	Statistic	*P* value	FDR
Gene	DESeq2 interaction	*MCM6*	Log_2_FC = 0.212	0.136	1.000
Gene	DESeq2 interaction	*CHEK1*	Log_2_FC = 0.148	0.171	1.000
Gene	DESeq2 interaction	*MCM3*	Log_2_FC = 0.267	0.192	1.000
Gene	DESeq2 interaction	*MCM4*	Log_2_FC = 0.212	0.234	1.000
Gene	DESeq2 interaction	*CDC6*	Log_2_FC = 0.175	0.284	1.000
Gene	DESeq2 interaction	*E2F1*	Log_2_FC = 0.284	0.430	1.000
Gene	DESeq2 interaction	*MCM5*	Log_2_FC = 0.350	0.433	1.000
Gene	DESeq2 interaction	*MCM7*	Log_2_FC = 0.237	0.441	1.000
Gene	DESeq2 interaction	*CDC25A*	Log_2_FC = 0.133	0.450	1.000
Gene	DESeq2 interaction	*MCM2*	Log_2_FC = 0.196	0.639	1.000
Pathway	GSEA (interaction-ranked)	HALLMARK_E2F_TARGETS	NES = 2.58	2.4 × 10^−23^	1.2 × 10^−21^
Pathway	GSEA (interaction-ranked)	HALLMARK_G2M_CHECKPOINT	NES = 2.44	2.9 × 10^−19^	7.1 × 10^−18^
Pathway	GSVA + limma factorial	HALLMARK_E2F_TARGETS	LogFC = 0.301	0.189	0.984
Pathway	GSVA + limma factorial	HALLMARK_G2M_CHECKPOINT	LogFC = 0.289	0.157	0.984

### Connectivity mapping identifies compounds associated with the female GDM transcriptional signature

To identify compounds associated with transcriptional patterns similar or dissimilar to the female GDM signature, connectivity mapping was performed using the L1000FWD platform, which compares an input gene-expression signature against a large reference collection of perturbation-induced transcriptional profiles (Fig. 9). The analysis identified several compounds exhibiting negative connectivity scores, indicating transcriptional signatures that opposed the observed female GDM expression profile. Among the highest-ranked negatively connected compounds was the CDK4/6 inhibitor *Palbociclib* (connectivity score = −0.40). Additional negatively connected compounds included *Naltrexone* and *FCCP*. These compounds were identified on the basis of transcriptional similarity alone, and no conclusions regarding therapeutic efficacy can be inferred from the present analysis. Connectivity mapping also identified several compounds with positive connectivity scores, indicating greater similarity to the female GDM transcriptional signature. These included *Chlorambucil*, *Cytarabine*, and *Carmofur* (connectivity score > 0.30). The observed connectivity patterns suggest that the female GDM transcriptional profile shares features with a range of experimentally induced transcriptional states represented within the LINCS reference database. Collectively, the L1000FWD analysis identified candidate compounds associated with either opposing or similar transcriptional signatures and provides a framework for future experimental evaluation of the pathways highlighted by the preceding transcriptomic and regulatory-network analyses.

**Fig. 9.**
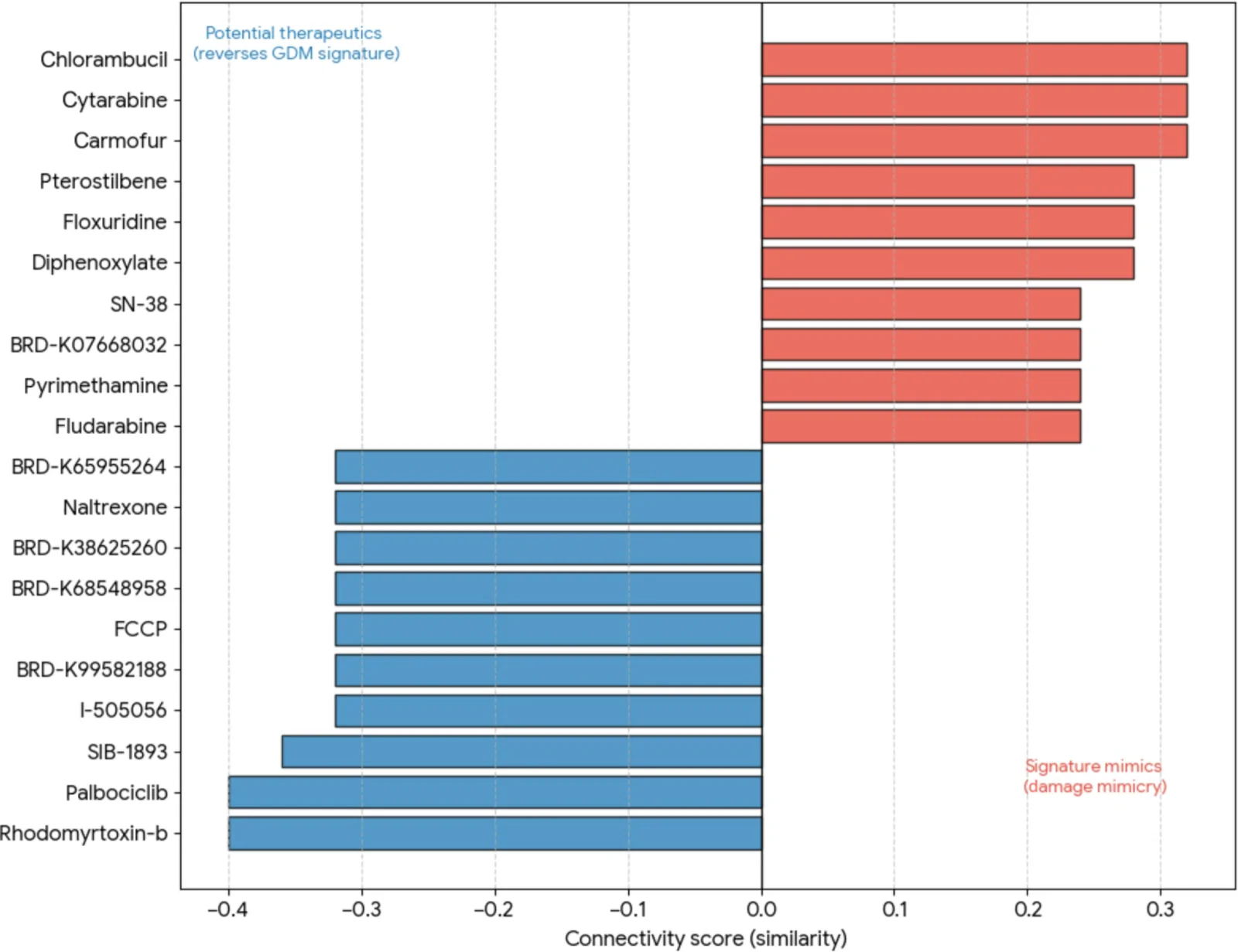
Connectivity mapping analysis of the female GDM transcriptional signature. Connectivity scores derived from the L1000FWD platform comparing the female GDM gene-expression signature to perturbation-induced transcriptional profiles in the LINCS reference database. Negative connectivity scores (blue bars) indicate compounds associated with transcriptional signatures opposing the female GDM profile, whereas positive connectivity scores (red bars) indicate compounds associated with transcriptional signatures showing greater similarity to the observed expression pattern. Representative negatively connected compounds include *Palbociclib*, *Naltrexone*, and *FCCP*, while positively connected compounds include *Chlorambucil*, *Cytarabine*, and *Carmofur*. Connectivity scores reflect transcriptomic similarity and do not constitute evidence of therapeutic efficacy or mechanistic validation.

## Discussion

In the present study, we identified marked sex-dependent differences in the transcriptomic responses of neonatal ECFCs to maternal metabolic dysfunction. Female ECFCs exhibited a substantially larger transcriptional response than male cells, characterized by enrichment of cell-cycle-associated pathways and recurrent prioritization of E2F-family regulators across multiple complementary computational methods applied to the same transcriptomic dataset. In contrast, the corresponding male response was comparatively limited and was associated with a distinct set of transcriptional changes, including differential expression of *AKT3*-associated pathways. Together, these findings indicate that male and female fetal vascular progenitors do not respond identically to maternal metabolic dysfunction and instead engage distinct transcriptional programs following GDM exposure.

A notable finding of this study was that these sex-dependent signatures were not apparent when male and female samples were analyzed together. An unstratified analysis of the complete 60-sample cohort identified no significantly differentially expressed genes, whereas direct comparison of male and female fold-change estimates revealed minimal concordance between the sexes. Similarly, modeling sex as a covariate rather than as a stratification variable did not recover the female-specific transcriptional patterns identified in the sex-stratified analyses. These observations suggest that biologically relevant features of the GDM-associated ECFC response may be obscured when fetal sex is treated solely as an adjustment factor rather than as a primary biological variable. A similar principle has recently been reported in placental transcriptomic studies, where fetal-sex stratification revealed differential-expression patterns that were not apparent in pooled analyses [13]. Together, these findings reinforce the value of sex-stratified investigation when examining fetal responses to adverse intrauterine environments.

An important feature of the present study is that the ECFCs were isolated from neonatal cord blood and cultured through multiple passages prior to transcriptomic analysis [18]. Consequently, the transcriptional differences observed here cannot be attributed to an ongoing maternal signal at the time of measurement. Rather, they reflect persistent differences in cellular state that remain detectable after removal from the intrauterine environment. Although clinical outcomes were not assessed, the persistence of these molecular signatures is consistent with the broader concept of developmental programming, in which environmental exposures during critical developmental windows induce long-lasting alterations in cellular function. Such persistent molecular differences represent the type of cellular phenomenon predicted by the DOHaD framework [49], even though downstream health outcomes were not evaluated in the present study.

Multiple complementary computational methods converged on an E2F-associated regulatory framework in female GDM-exposed ECFCs. Cell-cycle-associated pathways were repeatedly enriched by GSEA and GSVA, E2F-family transcription factors emerged as the highest-ranked candidates in motif and transcription-factor enrichment analyses, and activity inference consistently prioritized E2F-family regulators. Notably, E2F1 transcript abundance remained relatively stable despite enrichment of E2F-associated pathways and coordinated expression changes among E2F-associated target genes. This disconnect between transcription factor expression and inferred regulatory activity is not uncommon. Correlation between transcription factor expression and the expression of downstream target genes is frequently weak because transcription factor function is influenced by regulatory mechanisms that extend beyond transcript abundance alone, including posttranscriptional regulation, protein–protein interactions, posttranslational modification, epigenetic regulation, and changes in subcellular localization [50,51]. Consequently, transcription factor activity is often inferred from the coordinated behavior of downstream target genes rather than from transcription factor mRNA abundance alone [52,53]. A comparable uncoupling of mRNA abundance from functional output has been reported directly in GDM tissue: Alternative polyadenylation was found to suppress protein translation from otherwise stable mRNA transcripts of metabolic and inflammatory genes in GDM adipose tissue, independent of any change in transcript abundance [54], a pattern conceptually consistent with the transcription-activity discordance observed for E2F1 in the present study.

Several observations converged on the same regulatory framework. Members of the E2F-associated target network, including *MCM2–7*, *CDC6*, *CDC25A*, and *CHEK1*, exhibited coordinated increases in expression, and E2F-family regulators were independently prioritized across iRegulon, ChEA3, X2K, and decoupleR analyses. The corresponding gene-level expression changes were modest, suggesting that the signal is more readily detectable at the level of coordinated pathways and regulatory networks than at individual genes. More broadly, these observations align with a principle increasingly recognized in systems biology: Biologically meaningful regulatory changes may emerge most clearly at the level of coordinated gene networks even when individual transcriptional changes are relatively small. Although the present data cannot determine the mechanism underlying the apparent disconnect between *E2F1* transcript abundance and E2F-associated target-gene expression, they are consistent with altered E2F-associated regulatory activity occurring in the absence of marked changes in *E2F1* mRNA levels.

A sex × GDM interaction test directly addressed whether the sex-dependent transcriptional response described throughout this study reflects a statistically significant interaction, rather than merely divergent significance patterns arising from separate sex-stratified analyses. No individual gene reached FDR-corrected significance for the sex × GDM interaction term, including each of the core E2F-associated target genes examined above. However, GSEA performed on genes ranked by the interaction statistic identified E2F-target and G2M-checkpoint gene sets as the 2 most significant sex-differential pathways genome-wide, alongside several immune- and inflammation-related pathways showing significant negative interaction enrichment. A complementary factorial model applied to GSVA pathway scores did not independently reproduce this result, although the direction of the interaction effect was consistent across both approaches. This pattern mirrors the broader structure of evidence reported throughout this study: Individual-gene effects are modest and difficult to resolve at the present sample size, while coordinated, pathway-level effects are more readily detected when evidence is aggregated across many genes simultaneously. We consider the pathway-level interaction result the more statistically defensible evidence that the transcriptional response to GDM differs significantly by fetal sex, while acknowledging that this result was not independently confirmed by every analytical approach, and that confirmation in larger, independent cohorts will be needed to establish its robustness.

The kinase enrichment analyses provide additional biological context for this regulatory framework. Among the highest-ranked kinases were *ATR*, *ATM*, *CHEK2*, and *CDK1/2*, all of which are well-established components of cell-cycle control and DNA-damage response pathways. Their recurrent prioritization across independent analyses is consistent with the enrichment of DNA replication- and cell-cycle-associated pathways identified in female GDM ECFCs. The MAPK and phosphatidylinositol 3-kinase (PI3K)–Akt pathways, which interact extensively with cell-cycle signaling, have also been identified as important convergence points linking metabolic dysfunction to broader disease processes [12]. Within this context, the kinase-associated signaling pathways prioritized here provide a biologically plausible framework through which maternal metabolic dysfunction may be associated with sex-dependent transcriptional responses. Similarly, estrogen–MAPK signaling crosstalk has previously been proposed as a contributor to sex-specific responses to metabolic stress [12]. Although this possibility was not directly examined in the present study, it represents one potential explanation for the sex-dependent transcriptional patterns observed here.

The functional findings reported in the external datasets do not map directly onto the cell-cycle-associated transcriptional signature identified here. O’Neill *et al*. [47] reported reduced net proliferation despite enrichment of E2F-target genes, whereas the Tokic *et al*. [48] analysis found that GDM reduced proliferation but increased network formation specifically in female feto-placental endothelial cells, which the authors interpreted as a shift toward a more migratory endothelial phenotype. It is important to note the scope of each comparator: O’Neill *et al*. [47] did not record donor sex and therefore cannot validate the sex-specific direction of the present findings, serving only as sex-agnostic corroboration of E2F-associated pathway involvement in GDM-exposed ECFC, and Tokic *et al*. [48] examined differentiated feto-placental endothelial cells rather than circulating ECFC progenitors, representing a related but biologically distinct population. These observations are broadly compatible with, rather than contradictory to, the transcriptional patterns observed in the present study. A coordinated cell-cycle-associated transcriptional program does not necessarily predict uniform changes across all proliferation- or angiogenesis-related phenotypes, and the comparator studies assessed functional endpoints such as net proliferation and migration rather than pathway-level transcriptional organization. Notably, Chen *et al*. [55] also reported that individual differentially expressed genes did not survive Benjamini–Hochberg correction in their sex-stratified analysis and concluded that fetal-sex stratification revealed biological responses that would otherwise remain undetected. This methodological observation is directly consistent with our own findings that biologically meaningful transcriptional signatures emerged only when fetal sex was treated as a primary analytical variable rather than incorporated solely as a statistical covariate. Independent functional support for the male-specific AKT3-associated signature identified in the present study comes from a separate study reporting impaired insulin-induced vasodilation and deficient AKT phosphorylation in fetoplacental vessels from GDM pregnancies, mediated through the PI3K/AKT/eNOS axis [56]. This study did not stratify or report outcomes by fetal sex and therefore cannot confirm the sex-specific direction of our finding; it nonetheless provides independent, protein-level functional evidence that AKT signaling is disrupted in GDM-exposed fetal vasculature, consistent with the pathway identified computationally in male ECFCs here.

Concurrent with the E2F-associated transcriptional signature, we observed reduced enrichment of several pathways and regulators associated with inflammatory, vascular, and reparative functions, including interleukin-6 (IL-6)–JAK–STAT3 signaling, *IRF1*, *FOXF1*, and angiogenesis-related pathways. This pattern is consistent with the broader sex-dependent transcriptional reorganization identified through RRHO analysis, which demonstrated limited concordance between male and female GDM-associated responses. However, these observations should not be interpreted as evidence of discrete biological states or independent regulatory modules, as the rank-based approaches used here do not establish mechanistic separation between these processes.

Connectivity mapping identified several compounds with transcriptional profiles opposing the female GDM signature, including the CDK4/6 inhibitor *Palbociclib*. Although connectivity scores do not provide evidence of therapeutic efficacy or mechanistic validation, the emergence of a cell-cycle-directed compound among the highest-ranked negatively connected perturbations is broadly consistent with the enrichment of cell-cycle-associated pathways and E2F-associated regulatory programs identified throughout this study. These observations should be regarded as hypothesis-generating and may help prioritize future experimental investigation.

The convergent nature of the pathway, network, kinase, and transcription-factor analyses strengthens confidence in the overall regulatory framework identified here. At the same time, some important considerations should inform interpretation of the findings. First, all analyses were performed using a single publicly available transcriptomic dataset, and the regulatory relationships identified here were inferred computationally rather than validated experimentally. Although multiple complementary computational methods converged on similar biological themes, confirmation through protein-level measurements, functional perturbation studies, and independent cohorts will be important. Second, sex stratification necessarily reduced the effective sample size (Table S2) within each comparison group (*n* = 10), limiting statistical power at the level of individual genes. Accordingly, our conclusions rely primarily on convergent evidence from pathway enrichment, regulatory-network reconstruction, and transcription-factor-activity inference rather than large effects at individual loci. Potentially relevant clinical variables, including maternal age, gestational age at delivery, mode of delivery, insulin treatment status, and maternal ethnicity, were unavailable for adjustment and may have contributed to some of the observed transcriptomic patterns. Detailed clinical and demographic metadata beyond donor identified, maternal metabolic group, and fetal sex were not provided in the public data deposition underlying this study: Consequently, a baseline clinical characteristics table could not be constructed as a result.

Taken together, our findings indicate that fetal sex is an important determinant of the endothelial progenitor response to maternal metabolic dysfunction. Across multiple analytical approaches, female GDM-exposed ECFCs exhibited a larger and more coordinated transcriptional response than male cells, characterized by enrichment of cell-cycle-associated pathways and recurrent prioritization of E2F-family regulators. These observations support the existence of sex-dependent regulatory programs associated with fetal vascular responses to GDM and highlight the value of treating fetal sex as a primary biological variable in studies of developmental programming. The E2F-associated regulatory framework identified here should be regarded as a candidate mechanism warranting further investigation in independent cohorts and experimental systems, particularly with respect to its potential role in shaping long-term vascular adaptation to adverse intrauterine metabolic environments (Fig. 10).

**Fig. 10.**
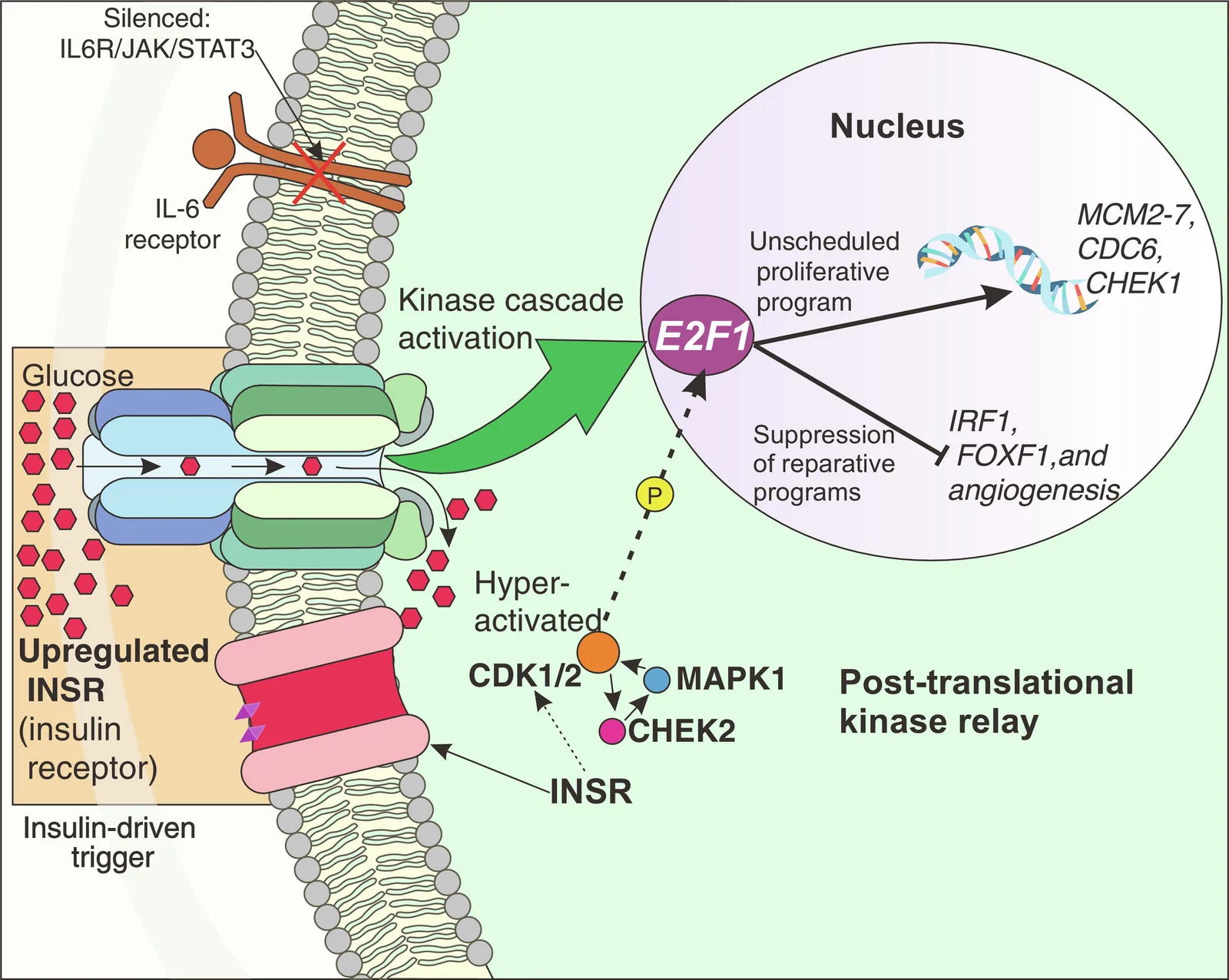
A proposed model linking GDM-associated hyperglycemia to E2F-associated transcriptional reprogramming in female ECFCs. Elevated glucose flux is hypothesized to up-regulate the insulin receptor (INSR), potentially triggering a kinase cascade involving CDK1/2, MAPK1, and CHEK2 that may posttranslationally activate E2F1, consistent with the transcriptional stability of E2F1 mRNA observed in this dataset. This proposed activation is associated with 2 divergent downstream transcriptional signatures identified in the present data: coordinated up-regulation of DNA replication and checkpoint genes (MCM2–7, CDC6, CHEK1), and concurrent reduced enrichment of homeostatic and reparative gene programs, including IRF1, FOXF1, angiogenesis-related genes, and IL-6 receptor/JAK/STAT3 signaling. This model is derived entirely from transcriptome-wide association and pathway-level evidence and represents one plausible, hypothesis-generating interpretation. Each step in the proposed signaling sequence—insulin receptor engagement, kinase activation, and posttranslational E2F1 activation—is inferred computationally and has not been directly measured at the protein or signaling level; alternative upstream triggers or intermediate steps cannot be excluded from transcriptomic data alone.

## Conclusion

In summary, this study demonstrates that fetal sex is an important determinant of the endothelial progenitor response to maternal metabolic dysfunction. By integrating sex-stratified transcriptomic analysis with pathway enrichment, regulatory-network reconstruction, transcription-factor activity inference, and connectivity mapping, we identified marked differences in the molecular responses of male and female ECFCs to GDM exposure. Female ECFCs exhibited a substantially larger and more coordinated transcriptional response than male cells, characterized by enrichment of cell-cycle-associated pathways and recurrent prioritization of E2F-family regulators across multiple complementary computational methods applied to the same transcriptomic dataset. Importantly, these signatures were not apparent in pooled analyses, indicating that biologically relevant features of the GDM-associated response may be obscured when fetal sex is treated solely as a statistical covariate. This finding reinforces the value of sex stratification in developmental programming and fetal vascular biology. The convergence of pathway, network, kinase, and transcription-factor analyses supports the existence of a female-specific regulatory framework associated with GDM exposure while also highlighting important differences between male and female endothelial adaptation to maternal metabolic stress. Independent endothelial transcriptomic datasets provided biological context consistent with these findings but, as previously published results rather than independent reanalysis, do not constitute direct validation of the sex-specific regulatory framework described here. Although the present study does not establish causality, it consistently implicates E2F-associated regulatory activity as a candidate mechanism, contributing to the female transcriptional signature. More broadly, these findings extend current understanding of developmental programming, demonstrating that adverse intrauterine metabolic exposures may be associated with distinct regulatory responses in male and female fetal vascular progenitors. Future studies incorporating independent cohorts, protein-level validation, correlation-aware sensitivity analyses of the pathway-level results presented here, and functional perturbation approaches would be necessary to elucidate how the regulatory programs identified here contribute to long-term vascular adaptation and disease susceptibility in offspring exposed to gestational diabetes.

## Data Availability

The raw transcriptomic data analyzed in this study are available in the National Center for Biotechnology Information (NCBI) Sequence Read Archive (SRA) database under BioProject accession number PRJNA952510. These data were originally generated as part of a cord-blood ECFC cohort; a subset of 35 samples from this cohort was analyzed and reported by Weiss *et al*. [18] in their investigation of gestational weight gain and fetal endothelial function, while the present study utilizes the complete 60-sample cohort, stratified by fetal sex and maternal metabolic status (Lean, Overweight, and Gestational Diabetes Mellitus) as annotated in the associated BioSample records. The processed data and custom bioinformatic scripts used in this study are available in https://github.com/Maxwellgbaju/GDM_Transcriptome.

## References

[1] Sánchez-Garrido MA , García-Galiano D , Tena-Sempere M . Early programming of reproductive health and fertility: Novel neuroendocrine mechanisms and implications in reproductive medicine. Hum Reprod Update. 2022;28(3): 346–375. 10.1093/humupd/dmac005 PMC9071071 35187579

[2] Hales CN , Barker DJ . Type 2 (non-insulin-dependent) diabetes mellitus: The thrifty phenotype hypothesis. Diabetologia. 1992;35(7):595–601. 10.1007/BF00400248 1644236

[3] Barker DJ . Fetal origins of coronary heart disease. BMJ. 1995;311(6998):171–174. 10.1136/bmj.311.6998.171 PMC2550226 7613432

[4] Yajnik CS . Fetal programming of diabetes: Still so much to learn! Diabetes Care. 2010;33(5):1146–1148. 10.2337/dc10-0407 PMC2858193 20427687

[5] Clifton VL , Murphy VE . Maternal asthma as a model for examining fetal sex-specific effects on maternal physiology and placental mechanisms that regulate human fetal growth. Placenta. 2004;25(Suppl):S45–S52. 10.1016/j.placenta.2004.01.004 15033307

[6] Clifton VL , Vanderlelie J , Perkins AV . Increased anti-oxidant enzyme activity and biological oxidation in placentae of pregnancies complicated by maternal asthma. Placenta. 2005;26(10):773–779. 10.1016/j.placenta.2004.10.018 16226128

[7] Stark MJ , Dierkx L , Clifton VL , Wright IMR . Alterations in the maternal peripheral microvascular response in pregnancies complicated by preeclampsia and the impact of fetal sex. J Soc Gynecol Investig. 2006;13(8):573–578. 10.1016/j.jsgi.2006.06.006 17055308

[8] Stark MJ , Clifton VL , Wright IMR . Neonates born to mothers with preeclampsia exhibit sex-specific alterations in microvascular function. Pediatr Res. 2009;65(3):292–295. 10.1203/pdr.0b013e318193edf1 19391250

[9] Stark MJ , Clifton VL , Wright IMR . Microvascular flow, clinical illness severity and cardiovascular function in the preterm infant. Arch Dis Child Fetal Neonatal Ed. 2008;93(4):F271–F274. 10.1136/adc.2007.123539 18285379

[10] Stark MJ , Clifton VL , Wright IMR . Sex-specific differences in peripheral microvascular blood flow in preterm infants. Pediatr Res. 2008;63(4):415–419. 10.1203/01.pdr.0000304937.38669.63 18356749

[11] Clifton VL . Review: Sex and the human placenta: Mediating differential strategies of fetal growth and survival. Placenta. 2010;31(Suppl):S33–S39. 10.1016/j.placenta.2009.11.010 20004469

[12] Onisiforou A , Zanos P . One path, two solutions: Network-based analysis identifies targetable pathways for the treatment of comorbid type II diabetes and neuropsychiatric disorders. Comput Struct Biotechnol J. 2024;23:3610–3624. 10.1016/j.csbj.2024.10.011 PMC11530817 39493502

[13] Smith MD , Plaisier S , Breen J , Bogias KJ , Jankovic-Karasoulos T , McCullough D , McAninch D , Arthurs AL , Wilson MA , Pillman KA , et al. Sex-specific placental transcriptome alterations in late-onset preeclampsia reveal male-biased immune and metabolic dysregulation. Biol Sex Differ. 2025;17(1):8. 10.1186/s13293-025-00781-w PMC12809948 41444673

[14] Parrettini S , Caroli A , Torlone E . Nutrition and metabolic adaptations in physiological and complicated pregnancy: Focus on obesity and gestational diabetes. Front Endocrinol. 2020;11:Article 611929. 10.3389/fendo.2020.611929 PMC7793966 33424775

[15] Strutz J , Cvitic S , Hackl H , Kashofer K , Appel HM , Thüringer A , Desoye G , Koolwijk P , Hiden U . Gestational diabetes alters microRNA signatures in human feto-placental endothelial cells depending on fetal sex. Clin Sci. 2018;132(22): 2437–2449. 10.1042/CS20180825 30389858

[16] Shook LL , White F , Acharya KD , Torres-Bigio S , Ibanez-Pintor L , Han D , Aguet F , Ardlie KG , Florez JC , Bouchard L , et al. Fetal sex-specific differences in the placental transcriptome of gestational diabetes. Sci Rep. 2026;16(1):9288. 10.1038/s41598-026-39975-6 PMC13000196 41699151

[17] Weiss E , Leopold-Posch B , Schrüfer A , Cvitic S , Hiden U . Fetal sex and maternal fasting glucose affect neonatal cord blood-derived endothelial progenitor cells. Pediatr Res. 2022;92(6):1590–1597. 10.1038/s41390-022-01966-4 PMC9771817 35184136

[18] Weiss E , Schrüfer A , Tocantins C , Diniz MS , Novakovic B , van Bergen AS , Kulovic-Sissawo A , Saffery R , Boon RA , Hiden U . Higher gestational weight gain delays wound healing and reduces expression of long non-coding RNA KLRK1-AS1 in neonatal endothelial progenitor cells. J Physiol. 2023;601(17):3961–3974. 10.1113/JP284871 PMC10952284 37470310

[19] Andrews S. FastQC: A quality control tool for high throughput sequence data. Babraham Bioinformatics. 2010.

[20] Bolger AM , Lohse M , Usadel B . Trimmomatic: A flexible trimmer for Illumina sequence data. Bioinformatics. 2014;30(15):2114–2120. 10.1093/bioinformatics/btu170 PMC4103590 24695404

[21] Kim D , Paggi JM , Park C , Bennett C , Salzberg SL . Graph-based genome alignment and genotyping with HISAT2 and HISAT-genotype. Nat Biotechnol. 2019;37(8):907–915. 10.1038/s41587-019-0201-4 PMC7605509 31375807

[22] Li H , Handsaker B , Wysoker A , Fennell T , Ruan J , Homer N , Marth G , Abecasis G , Durbin R , 1000 Genome Project Data Processing Subgroup . The Sequence Alignment/Map format and SAMtools. Bioinformatics. 2009;25(16):2078–2079. 10.1093/bioinformatics/btp352 PMC2723002 19505943

[23] Broad Institute. Picard Tools. [accessed 4 Feb 2026] http://broadinstitute.github.io/picard/

[24] Love MI , Huber W , Anders S . Moderated estimation of fold change and dispersion for RNA-seq data with DESeq2. Genome Biol. 2014;15(12):550. 10.1186/s13059-014-0550-8 PMC4302049 25516281

[25] Plaisier SB , Taschereau R , Wong JA , Graeber TG . Rank-rank hypergeometric overlap: Identification of statistically significant overlap between gene-expression signatures. Nucleic Acids Res. 2010;38(17):Article e169. 10.1093/nar/gkq636 PMC2943622 20660011

[26] Rosenblatt JD, Stein JL. RRHO: Test overlap using the rank-rank hypergeometric overlap test. R package version 1.42.0. 2014.

[27] Liao Y , Wang J , Jaehnig EJ , Shi Z , Zhang B . WebGestalt 2019: Gene set analysis toolkit with revamped UIs and APIs. Nucleic Acids Res. 2019;47(W1):W199–W205. 10.1093/nar/gkz401 PMC6602449 31114916

[28] Benjamini Y , Hochberg Y . Controlling the false discovery rate: A practical and powerful approach to multiple testing. J R Stat Soc Ser B Stat Methodol. 1995;57(1):289–300.

[29] Wu T , Hu E , Xu S , Chen M , Guo P , Dai Z , Feng T , Zhou L , Tang W , Li Z , et al. clusterProfiler 4.0: A universal enrichment tool for interpreting omics data. Innovation. 2021;2(3): Article 100141. 10.1016/j.xinn.2021.100141 PMC8454663 34557778

[30] Hänzelmann S , Castelo R , Guinney J . GSVA: Gene set variation analysis for microarray and RNA-seq data. BMC Bioinformatics. 2013;14:7. 10.1186/1471-2105-14-7 PMC3618321 23323831

[31] Liberzon A , Birger C , Thorvaldsdóttir H , Ghandi M , Mesirov JP , Tamayo P . The Molecular Signatures Database (MSigDB) hallmark gene set collection. Cell Syst. 2015;1(6): 417–425. 10.1016/j.cels.2015.12.004 PMC4707969 26771021

[32] Ritchie ME , Phipson B , Wu D , Hu Y , Law CW , Shi W , Smyth GK . limma powers differential expression analyses for RNA-sequencing and microarray studies. Nucleic Acids Res. 2015;43(7):Article e47. 10.1093/nar/gkv007 PMC4402510 25605792

[33] Korotkevich G, Sukhov V, Budin N, Shpak B, Artyomov MN, Sergushichev A. Fast gene set enrichment analysis. bioRxiv. 2021. 10.1101/060012

[34] Dolgalev I. msigdbr rds. Version 2026.1. Zenodo. 2026. 10.5281/zenodo.18968178

[35] Janky R , Verfaillie A , Imrichová H , Van de Sande B , Standaert L , Christiaens V , Hulselmans G , Herten K , Sanchez MN , Potier D , et al. iRegulon: From a gene list to a gene regulatory network using large motif and track collections. PLOS Comput Biol. 2014;10(7):Article e1003731. 10.1371/journal.pcbi.1003731 PMC4109854 25058159

[36] Shannon P , Markiel A , Ozier O , Baliga NS , Wang JT , Ramage D , Amin N , Schwikowski B , Ideker T . Cytoscape: A software environment for integrated models of biomolecular interaction networks. Genome Res. 2003;13(11):2498–2504. 10.1101/gr.1239303 PMC403769 14597658

[37] ENCODE Project Consortium. An integrated encyclopedia of DNA elements in the human genome. Nature. 2012;489(7414): 57–74. 10.1038/nature11247 PMC3439153 22955616

[38] Chen EY , Xu H , Gordonov S , Lim MP , Perkins MH , Ma’ayan A . Expression2Kinases: mRNA profiling linked to multiple upstream regulatory layers. Bioinformatics. 2012;28(1):105–111. 10.1093/bioinformatics/btr625 PMC3244772 22080467

[39] Keenan AB , Torre D , Lachmann A , Leong AK , Wojciechowicz ML , Utti V , Jagodnik KM , Kropiwnicki E , Wang Z , Ma’ayan A . ChEA3: Transcription factor enrichment analysis by orthogonal omics integration. Nucleic Acids Res. 2019;47(W1):W212–W224. 10.1093/nar/gkz446 PMC6602523 31114921

[40] Kuleshov MV , Xie Z , London ABK , Yang J , Evangelista JE , Lachmann A , Shu I , Torre D , Ma’ayan A . KEA3: Improved kinase enrichment analysis via data integration. Nucleic Acids Res. 2021;49(W1):W304–W316. 10.1093/nar/gkab359 PMC8265130 34019655

[41] R Core Team. *R: A language and environment for statistical computing*. Vienna: R Foundation for Statistical Computing; 2023.

[42] Müller-Dott S , Tsirvouli E , Vazquez M , Ramirez Flores RO , Badia-I-Mompel P , Fallegger R , Türei D , Lægreid A , Saez-Rodriguez J . Expanding the coverage of regulons from high-confidence prior knowledge for accurate estimation of transcription factor activities. Nucleic Acids Res. 2023;51(20):10934–10949. 10.1093/nar/gkad841 PMC10639077 37843125

[43] Badia-I-Mompel P , Vélez Santiago J , Braunger J , Geiss C , Dimitrov D , Müller-Dott S , Taus P , Dugourd A , Holland CH , Ramirez Flores RO , et al. decoupleR: Ensemble of computational methods to infer biological activities from omics data. Bioinform Adv. 2022;2(1):Article vbac016. 10.1093/bioadv/vbac016 PMC9710656 36699385

[44] Durinck S , Spellman PT , Birney E , Huber W . Mapping identifiers for the integration of genomic datasets with the R/Bioconductor package biomaRt. Nat Protoc. 2009;4(8):1184–1189. 10.1038/nprot.2009.97 PMC3159387 19617889

[45] Durinck S , Moreau Y , Kasprzyk A , Davis S , De Moor B , Brazma A , Huber W . BioMart and Bioconductor: A powerful link between biological databases and microarray data analysis. Bioinformatics. 2005;21(16):3439–3440. 10.1093/bioinformatics/bti525 16082012

[46] Kinsella RJ , Kähäri A , Haider S , Zamora J , Proctor G , Spudich G , Almeida-King J , Staines D , Derwent P , Kerhornou A , et al. Ensembl BioMarts: A hub for data retrieval across taxonomic space. Database. 2011;2011:Article bar030. 10.1093/database/bar030 PMC3170168 21785142

[47] O’Neill KM , Edgar KS , Pun SH , Campbell DC , Toh T , Wong XN , Botezatu B , Kandel J , McCoy U , Nicell J , et al. Restoration of NOX4 signalling reverses endothelial colony-forming cell angiogenic dysfunction associated with experimental and clinical diabetes. Stem Cell Res Ther. 2025;16(1):275. 10.1186/s13287-025-04393-4 PMC12131569 40457435

[48] Tokic S , Schlagenhauf A , Dohr KA , Desoye G , Hiden U . Feto-placental endothelial cells of female neonates are more susceptible to gestational diabetes-induced changes. Histochem Cell Biol. 2025;163(1):107. 10.1007/s00418-025-02433-x PMC12647237 41288732

[49] Wang Z , Lachmann A , Keenan AB , Ma’ayan A . L1000FWD: Fireworks visualization of drug-induced transcriptomic signatures. Bioinformatics. 2018;34(12):2150–2152. 10.1093/bioinformatics/bty060 PMC6454499 29420694

[50] Gluckman PD , Hanson MA , Cooper C , Thornburg KL . Effect of in utero and early-life conditions on adult health and disease. N Engl J Med. 2008;359(1):61–73. 10.1056/NEJMra0708473 PMC3923653 18596274

[51] Filtz TM , Vogel WK , Leid M . Regulation of transcription factor activity by interconnected post-translational modifications. Trends Pharmacol Sci. 2014;35(2):76–85. 10.1016/j.tips.2013.11.005 PMC3954851 24388790

[52] Zaborowski AB , Walther D . Determinants of correlated expression of transcription factors and their target genes. Nucleic Acids Res. 2020;48(20):11347–11369. 10.1093/nar/gkaa927 PMC7672440 33104784

[53] Boorsma A , Lu X-J , Zakrzewska A , Klis FM , Bussemaker HJ . Inferring condition-specific modulation of transcription factor activity in yeast through regulon-based analysis of genomewide expression. PLOS ONE. 2008;3(9):Article e3112. 10.1371/journal.pone.0003112 PMC2518834 18769540

[54] Ma CZ , Brent MR . Inferring TF activities and activity regulators from gene expression data with constraints from TF perturbation data. Bioinformatics. 2021;37(9):1234–1245. 10.1093/bioinformatics/btaa947 PMC8189679 33135076

[55] Chen B , Chen X , Hu R , Li H , Wang M , Zhou L , Chen H , Wang J , Zhang H , Zhou X , et al. Alternative polyadenylation regulates the translation of metabolic and inflammation-related proteins in adipose tissue of gestational diabetes mellitus. Comput Struct Biotechnol J. 2024;23:1298–1310. 10.1016/j.csbj.2024.03.013 PMC10978812 38560280

[56] Hengst CM , Villar-Ballesteros ML , Brendel H , Giebe S , Brunssen C , Frühauf A , Birdir C , Taylor PD , Poston L , Morawietz H . Maternal gestational diabetes impairs fetoplacental insulin-induced vasodilation via AKT/eNOS pathway and reduces placental efficiency. Int J Mol Sci. 2025;26(23):11507. 10.3390/ijms262311507 PMC12692426 41373661

