# Supplementary material for: Sex-Stratified Transcriptomic Analysis of Cord-Blood Endothelial Colony-Forming Cells from Pregnancies Complicated by Gestational Diabetes
Source: Comput Struct Biotechnol J. 2026 Nov 6;35(1):0252. doi: 10.34133/csbj.0252 (PMC13740330; doi:10.34133/csbj.0252)
Supplement: Supplementary file 1 [file csbj.0252.f1.zip › Supplementary Figure S1.docx]

**Supplementary Figure S1. GSVA pathway activity scores for E2F Targets (A) and G2M Checkpoint (B) Hallmark gene sets, by fetal sex and GDM status.** Boxplots show single-sample GSVA enrichment scores for Female-Lean, Female-GDM, Male-Lean, and Male-GDM ECFCs (n = 10 per group). Horizontal lines indicate group medians, boxes indicate the interquartile range, and individual points represent single samples. In female ECFCs, GDM exposure was associated with a positive shift in enrichment scores for both pathways relative to Lean controls, consistent with the concordant upregulation of the corresponding gene-level signature described in the main text. In male ECFCs, GDM exposure was associated with a comparatively small negative shift for both pathways relative to Lean controls. These between-group differences did not reach statistical significance (Wilcoxon rank-sum test; Female GDM vs. Lean, E2F Targets P = 0.247; Male GDM vs. Lean, E2F Targets P = 0.796), consistent with the modest per-gene effect sizes underlying this signature and the limited statistical power at n = 10 per group. This figure is intended to illustrate the direction of the sex-dependent trend rather than to provide independent statistical confirmation of it.
