# Supplementary material for: Sex-Stratified Transcriptomic Analysis of Cord-Blood Endothelial Colony-Forming Cells from Pregnancies Complicated by Gestational Diabetes
Source: Comput Struct Biotechnol J. 2026 Nov 6;35(1):0252. doi: 10.34133/csbj.0252 (PMC13740330; doi:10.34133/csbj.0252)
Supplement: Supplementary file 1 [file csbj.0252.f1.zip › Supplementary Table S1.docx]

| Gene |  | baseMean | log_2_FC | lfcSE | stat | p-value | padj |
| --- | --- | --- | --- | --- | --- | --- | --- |
| MCM6 |  | 6729.08 | 0.112 | 0.077 | 1.453 | 0.146 | 1.000 |
| CDC25A |  | 2009.67 | 0.038 | 0.087 | 0.434 | 0.664 | 1.000 |
| MCM2 |  | 1104.72 | 0.093 | 0.188 | 0.497 | 0.619 | 1.000 |
| MCM3 |  | 7360.49 | 0.040 | 0.100 | 0.401 | 0.689 | 1.000 |
| MCM7 |  | 7598.61 | 0.110 | 0.139 | 0.792 | 0.428 | 1.000 |
| MCM4 |  | 7318.00 | 0.061 | 0.080 | 0.767 | 0.443 | 1.000 |
| CHEK1 |  | 4079.99 | 0.087 | 0.079 | 1.104 | 0.270 | 1.000 |
| CDC6 |  | 693.54 | 0.068 | 0.172 | 0.392 | 0.695 | 1.000 |
| MCM5 |  | 2278.34 | 0.075 | 0.200 | 0.372 | 0.710 | 1.000 |

**Supplementary Table S1: Differential expression statistics for E2F1-regulon target genes under a sex-covariate model (GDM vs. Lean, full 60-sample cohort)**
