# Supplementary material for: Sex-Stratified Transcriptomic Analysis of Cord-Blood Endothelial Colony-Forming Cells from Pregnancies Complicated by Gestational Diabetes
Source: Comput Struct Biotechnol J. 2026 Nov 6;35(1):0252. doi: 10.34133/csbj.0252 (PMC13740330; doi:10.34133/csbj.0252)
Supplement: Supplementary file 1 [file csbj.0252.f1.zip › Supplementary Table S2.docx]

| Contrast | Genes Tested | Nominal p < 0.05 | FDR padj < 0.05 | FDR padj < 0.10 | % Nominal Surviving FDR |
| --- | --- | --- | --- | --- | --- |
| Female: GDM vs Lean | 38,947 | 597 | 0 | 0 | 0% |
| Female: GDM vs Overweight | 38,947 | 583 | 0 | 0 | 0% |
| Female: Overweight vs Lean | 38,947 | 702 | 0 | 3 | 0% |
| Male: GDM vs Lean | 38,695 | 528 | 0 | 0 | 0% |
| Male: GDM vs Overweight | 38,695 | 749 | 2 | 2 | 0.3% |
| Male: Overweight vs Lean | 38,695 | 694 | 1 | 1 | 0.1% |
| Unstratified: GDM vs Lean | 41,758 | 695 | 0 | 1 | 0% |

**Supplementary Table S2: Nominal and FDR-adjusted differential expression across all contrasts**
